# Multiscale Engineering of Ion‐Conducting Gels for Sustainable Bioelectronic Systems

**DOI:** 10.1002/smtd.202501625

**Published:** 2025-11-19

**Authors:** Ji Hong Kim, Won Hyuk Choi, Jong Hwi Kim, Yoseph Park, Seonghwan Yun, Tae‐il Kim, Do Hwan Kim

**Affiliations:** ^1^ Department of Chemical Engineering Hanyang University Seoul 04763 Republic of Korea; ^2^ Institute of Nano Science and Technology Hanyang University Seoul 04763 Republic of Korea; ^3^ Clean‐Energy Research Institute Hanyang University Seoul 04763 Republic of Korea; ^4^ School of Chemical Engineering Sungkyunkwan University Suwon 16419 Republic of Korea

**Keywords:** adaptive diagnostics and therapeutics, closed‐loop bioelectronic systems, implantable devices, ion‐conducting gel, sustainable bioelectronics

## Abstract

Ion‐conducting gels are indispensable for bioelectronics, offering softness, high ionic conductivity, and biocompatibility. Nevertheless, sustaining robust performance under physiological conditions demands moving beyond isolated material or device innovations to a unified, multiscale design approach. At the material level, advances in polymer network engineering enable precise tuning of ion mobility, retention, and electrochemical stability, while simultaneously imparting mechanical toughness, hydration preservation, and self‐healing. At the device level, these gels are tailored for seamless electrode integration, ensuring high signal fidelity, low impedance, and stable ionic–electronic coupling under deformation. When integrated into closed‐loop architectures encompassing biosignal acquisition, signal processing, and feedback control, ion‐conducting gels evolve from passive conductors into active, reconfigurable elements within autonomous diagnostic and therapeutic systems. This review highlights the critical interplay of material design, device integration, and system‐level engineering in advancing long‐lived, sustainable bioelectronic technologies.

## Graphical abstract

This review highlights multiscale engineering strategies for ion‐conducting gels aimed at achieving sustainable bioelectronic systems. It systematically examines material‐level, device‐level, and system‐level approaches to ensure long‐term stability and functional integrity under physiological conditions, integrating molecular design, device engineering, and system architecture into a unified roadmap for next‐generation wearable and implantable bioelectronics.

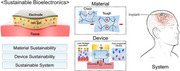

## 1 Introduction

The human body processes and transmits electrical signals via ionic currents, whereas artificial electronic systems rely primarily on electrons.^[^ ^1^, ^2^, ^3^ ^]^ At their junction, the human–machine interface, effective communication demands hybrid ionic‐electronic coupling to bridge these fundamentally different charge carriers.^[^ ^4^, ^5^, ^6^, ^7^ ^]^ This requirement fuels the need for implantable bioelectronic systems capable of precise biosignal sensing and active physiological feedback.

Bioelectronics is an interdisciplinary field merging biological systems with electronic technologies to detect, process, and modulate biological signals, with origins dating to the mid‐20th century.^[^ ^8^, ^9^, ^10^ ^]^ Early efforts centered on recording electrophysiological activity, electrocardiography (ECG), electroencephalography (EEG), and electromyography (EMG), thereby laying the groundwork for machine–tissue interfaces. Over time, the field moved beyond simple device application toward integrating biological principles within electronic platforms to enable bidirectional communication, biomimetic functionality, and active physiological modulation.

**Figure 1:**
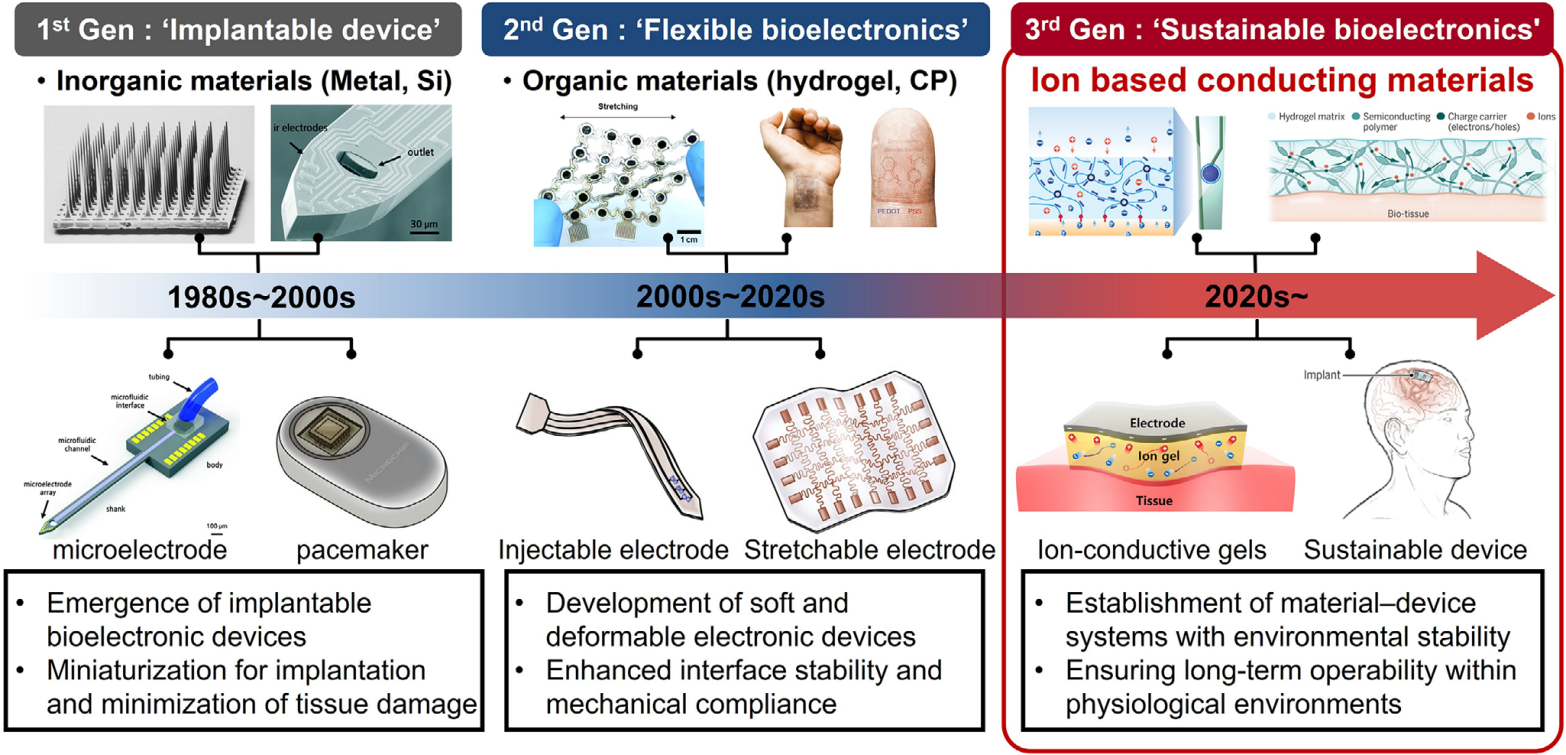
Timeline of bioelectronics evolution driven by advances in material platforms. Reproduced with permission.^[^ ^23^, ^52^ ^]^ Copyright 2020,2023, Wiley‐VCH. Reproduced with permission.^[^ ^26^ ^]^ Copyright 2025, Royal Society of Chemistry. Reproduced with permission.^[^ ^35^, ^39^, ^46^ ^]^ Copyright 2022,2024, American Association for the Advancement of Science.

**Figure** **1** charts the material‐driven evolution of bioelectronics. In the 1980s–1990s, advances in silicon electronics enabled rapid miniaturization and system integration, catalyzing implantable devices that could augment or replace physiological functions.^[^ ^11^, ^12^ ^]^ Representative examples include cochlear implants,^[^ ^13^ ^]^ deep‐brain stimulators,^[^ ^14^, ^15^ ^]^ and cardiac pacemakers^[^ ^16^ ^]^, which improved health through real‐time monitoring^[^ ^17^, ^18^, ^19^ ^]^ and targeted electrical stimulation.^[^ ^20^, ^21^, ^22^, ^23^ ^]^ Despite these successes, rigid inorganic devices^[^ ^24^, ^25^, ^26^, ^27^ ^]^ often exhibit severe mechanical mismatches with soft, dynamic tissues.^[^ ^28^, ^29^, ^30^ ^]^ Continuous deformation during normal physiology can provoke inflammation, tissue damage, poor electrical coupling, and shortened device lifetimes—highlighting fundamental limits in mechanical compatibility and biointegration. These limitations motivated a transition toward next‐generation platforms built from compliant materials.

Since the 2000s, flexible and stretchable electronics have emerged to address stiffness and biocompatibility constraints. The introduction of soft materials—organic semiconductors,^[^ ^31^, ^32^, ^33^, ^34^, ^35^ ^]^ conductive polymers such as PEDOT:PSS, and hydrogels^[^ ^36^, ^37^, ^38^, ^39^, ^40^, ^41^, ^42^, ^43^, ^44^, ^45^, ^46^ ^]^—expanded applications to skin‐integrated sensors (e‐skin), flexible neuromodulators, and implantable biosensors. In this review, “soft materials” refers to polymer‐based, tissue‐compliant materials in their working state that can be processed into conformal films and elastomeric structures for bioelectronic interfaces. We use this umbrella term to encompass organic semiconductors and conductive polymers that support electronic transport, as well as hydrogels and ionogels that support ionic transport, since these components are often co‐integrated to combine charge transduction with mechanical matching. When transport mechanisms are discussed, we explicitly distinguish electronic and ionic conductors. Improved mechanical compliance and conformability enabled stable operation under motion. However, hydrogel‐ and gel‐based conductors face important constraints in vivo. Hydration instability (evaporation, osmotic swelling, environmental perturbations) can drive volumetric fluctuations that undermine mechanical integrity and interfacial stability.^[^ ^47^, ^48^, ^49^, ^50^, ^51^ ^]^ Electrochemical instability under physiological conditions, via redox side reactions or doping‐level drift, can irreversibly alter conductivity and raise interfacial impedance^[^ ^52^, ^53^, ^54^ ^]^, accelerating electrode corrosion and causing signal‐transduction failure.^[^ ^55^ ^]^ These challenges complicate long‐term reliability, particularly for fully implantable systems operating in dynamic aqueous environments.

To overcome these limitations and achieve more seamless integration with living tissues, ion‐conducting gels have recently gained prominence as a materials platform for bioelectronics. These soft, polymeric networks entrap a liquid phase, typically water or ionic liquids, providing efficient ionic transport while preserving mechanical robustness. Beyond passive compliance, ion‐conducting gels emulate the body's ionic signaling, enabling biologically relevant sensing and transduction. At metal–electrode interfaces, they support electric double‐layer (EDL) formation that stabilizes electrochemical behavior and mediates efficient conversion between ionic and electronic currents.^[^ ^56^, ^57^, ^58^, ^59^ ^]^ Collectively, these attributes position ion‐conducting gels as a foundation for durable, high‐performance electrical interfaces with biological tissues.

Nevertheless, much current work optimizes isolated material properties rather than ensuring system‐level sustainability and reliability. Ion‐driven instabilities can still degrade devices and compromise biocompatibility over time. Accordingly, sustainable integration across materials, interfaces, device architectures, and system design under physiological conditions is essential. Previous reviews have typically emphasized either material designs, device architectures, or application demonstrations. While each perspective is informative, these strands are often treated separately, with limited linkage from composition and network design to interface metrics and to system operation under physiological variability. In contrast, our review adopts a platform‐based view anchored in ion‐conducting gels and carried consistently across materials, devices, and systems. We synthesize design rules that connect materials choices to electrochemical operating ranges and ionic transport, translate these rules into device‐level reliability and signal metrics, and integrate them with closed‐loop and lifetime considerations at the system level. This cross‐scale, design‐rule perspective complements prior surveys by providing a coherent scaffold for sustainable function in realistic biological environments.

In this review, sustainability denotes the capacity of ion‐conducting‐gel bioelectronics to deliver intended sensing or stimulation with acceptable risk over clinically relevant timescales in physiological environments while minimizing material and environmental burdens. We evaluate sustainability along four coupled dimensions: functional endurance that preserves electrical performance and signal fidelity under mechanical, thermal, and chemical stress; biosafety that limits adverse biological responses by controlling ion release and avoiding harmful reaction pathways within safe electrochemical operating ranges; resource efficiency that reduces operating power, streamlines scalable processing, and curtails waste across the device life; and verifiability that relies on standardized testing, transparent uncertainty reporting, and reproducible metrics to enable fair comparison across studies. Building on this premise, the following sections examine the key requirements for sustainable ion‐conducting gels in bioelectronics, linking materials chemistry to interface engineering, failure modes to reliability metrics, and device‐level performance to closed‐loop system operation.

**Figure 2:**
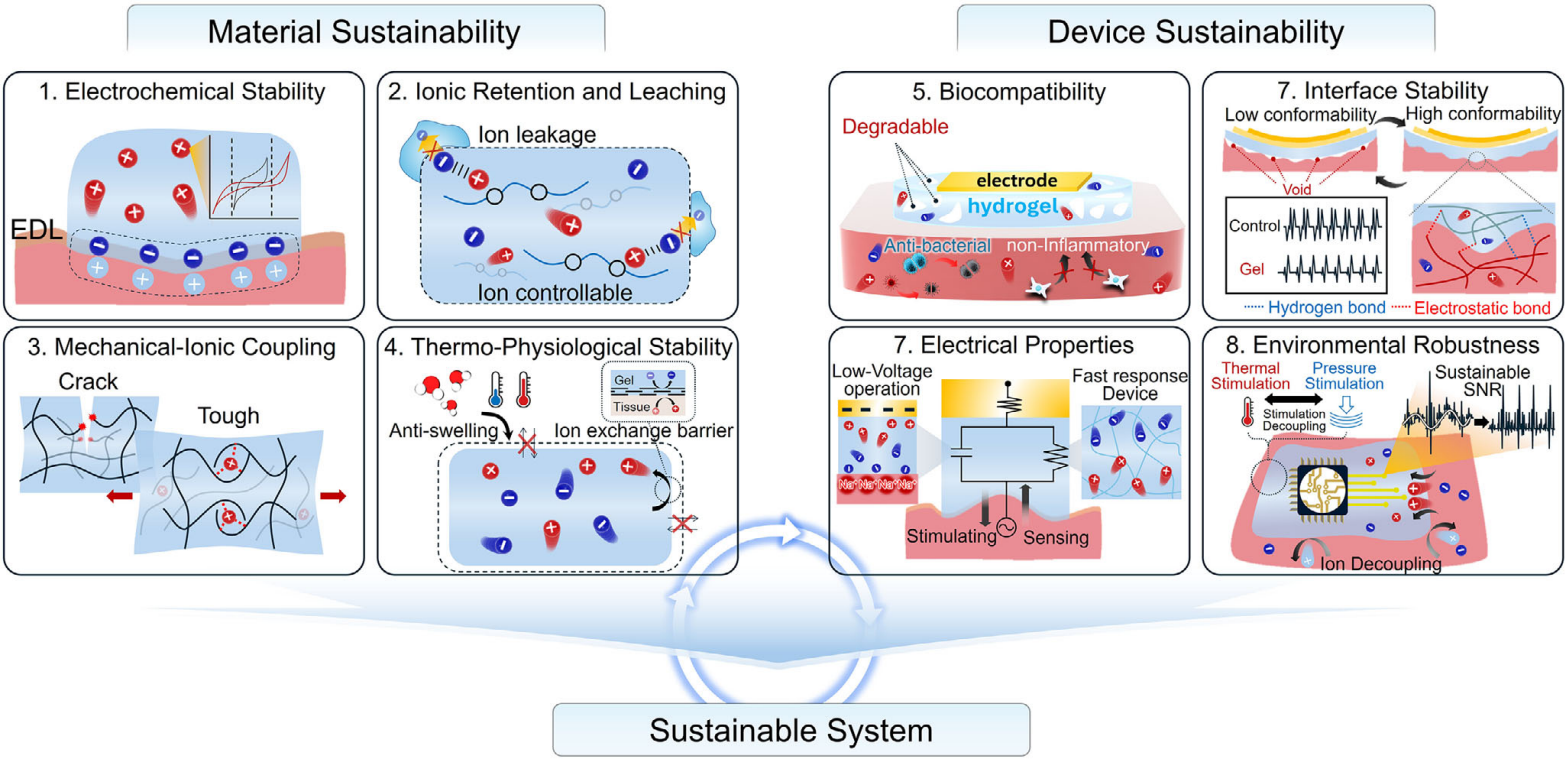
Properties for sustainable bioelectronics across the material (electrochemical stability; ionic retention and leaching; mechanical‐ionic coupling; and thermo‐physiological stability), device (biocompatibility; interface stability; electrical properties; environmental robustness), and system levels.

**Figure** **2** schematically presents the integrated system framework discussed in this review. First, we explore material sustainability, focusing on electrochemical stability, ionic retention and leaching resistance, mechanical robustness, and thermo‐physiological stability—key factors governing the intrinsic durability and functionality of ion‐conducting gels under physiological conditions. Next, we address device sustainability, emphasizing biocompatibility, interface stability, high‐performance electrical properties, and environmental robustness to ensure reliable operation and integration with biological systems. Finally, we discuss system sustainability, highlighting AI‐enabled closed‐loop platforms that pair durable materials and devices with lightweight edge inference and reinforcement learning for real‐time decoding and adaptation of biosignals within predefined safety limits, as well as AI‐based device‐health monitoring that tracks impedance and interface changes to maintain low‐voltage operation and contact quality; standardized testing and life‐cycle assessment anchor these controllers to reproducible metrics and environmental accountability. Together, these interconnected layers form a holistic framework for guiding the design and implementation of next‐generation bioelectronic technologies.

## 2 Material Sustainability of Ion‐Conducting Gels for Bioelectronics

Sustainable material design is fundamental to the development of implantable bioelectronic systems, where long‐term functional stability within the physiological environment is essential. In this section, we focus on the intrinsic stability and material sustainability of ion‐conducting gels, which serve as key components for reliable and long‐lasting bioelectronic interfaces. Specifically, we discuss how tailored material can mitigate degradation and ensure consistent device performance.

### 2.1 Electrochemical Stability

Implantable bioelectronic devices, particularly those employing electrical stimulation, have emerged as powerful platforms for real‐time physiological monitoring and therapeutic modulation. These systems interface directly with ionic environments within the human body, enabling the detection of endogenous electrophysiological signals or the delivery of artificial electrical cues to restore or modulate biological function. However, the application of external electrical fields within biological tissues presents significant electrochemical challenges. Uncontrolled redox reactions, ionic imbalances, local electrolysis,^[^ ^60^, ^61^, ^62^, ^63^, ^64^, ^65^ ^]^ pH shifts,^[^ ^66^, ^67^, ^68^ ^]^ gas evolution,^[^ ^69^, ^70^ ^]^ and protein denaturation can occur at the material–tissue interface, potentially leading to cellular damage and compromised device functionality. Therefore, ensuring electrochemical stability is essential for the safe and reliable operation of such devices under physiological conditions.

Conducting polymers and hydrogels have been widely employed in bioelectronics due to their excellent biocompatibility and mechanical softness. However, their intrinsically narrow electrochemical window limits their stability under electrical stimulation.^[^ ^71^, ^72^ ^]^ Even at low applied potential, these materials often undergo irreversible faradaic reactions at the bio‐interface, particularly during neural signal transmission. This charge injection mechanism can trigger undesirable electrochemical events, such as water electrolysis and electrode oxidation, leading to gas evolution and material degradation.^[^ ^73^, ^74^, ^75^ ^]^ These effects compromise the long‐term electrochemical stability of the device and may negatively impact its biocompatibility and functional integration with surrounding tissues.

Although the incorporation of salts, organic solvents, and ionic liquids can significantly broaden the electrochemical stability window of ion‐conducting gels, these modifications inherently carry risks to biocompatibility that must be addressed. For example, chaotropic anions are known to disrupt hydrogen‐bonding networks and destabilize protein structures, thereby potentially inducing protein denaturation or membrane perturbations at high local concentrations.^[^ ^76^ ^]^ Ionic liquids (ILs), while offering excellent electrochemical properties, have been documented to exert cytotoxic effects depending on their cation/anion combinations, alkyl chain lengths, and hydrophobicity.^[^ ^77^ ^]^ Moreover, uncontrolled leakage of mobile ions from the gel into the bloodstream can trigger inflammatory responses or cause cellular dysfunction, ultimately leading to tissue damage.^[^ ^78^ ^]^ Residual organic solvents may further amplify cytotoxicity if not fully removed or immobilized within the polymer matrix. To mitigate these risks, it is essential to 1) select ionic species with demonstrated low cytotoxicity (e.g., choline‐based or biomimetic ions), 2) minimize or fully remove residual organic solvents via purification or in situ crosslinking, 3) design immobilization or tethering strategies for mobile ions to prevent leakage, and 4) incorporate buffering or antioxidant moieties to neutralize any minor side reactions or reactive oxygen species. In this way, the expanded electrochemical window can be achieved without sacrificing safe long‐term bio integration.

**Figure 3:**
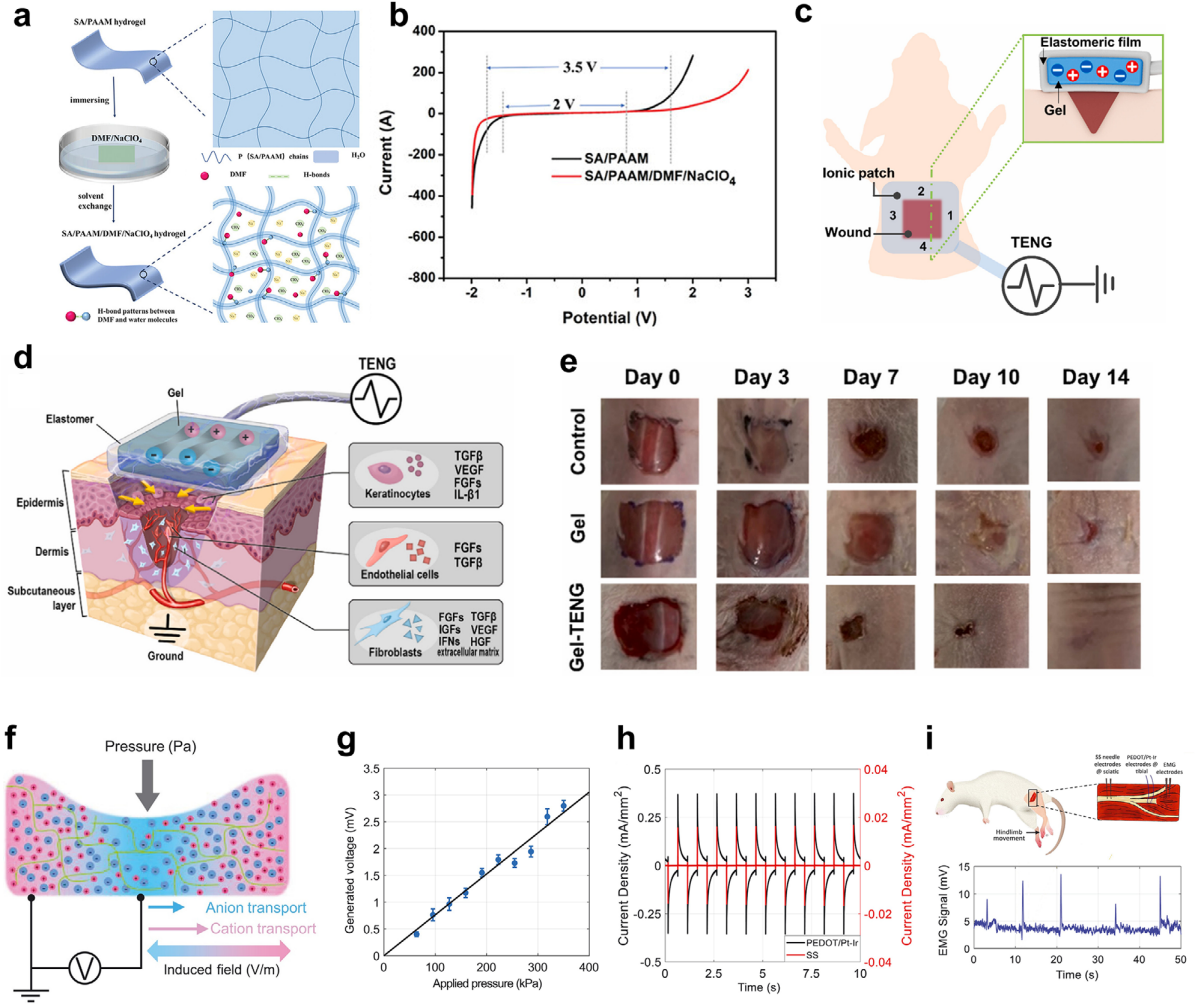
a) Material design and fabrication of a wide electrochemically stable window hydrogel. b) Comparison of CV curves between the conventional hydrogel and the ion‐incorporated hydrogel. c) In vivo wound healing test with an ionic patch on a full‐thickness wound, driven by TENG. d) Schematic illustration of organogel‐mediated charge injection into the wound site, promoting tissue regeneration under an electric field generated by an external self‐powered device. e) Time‐lapse photographs showing macroscopic wound recovery in untreated (control), only ionic patch (Gel), and ionic patch with TENG(Gel‐TENG). f) Illustration of asymmetric ion transport in a deformed polymer gel under pressure, resulting in a charge imbalance and a transient voltage signal. g) Peak generated voltage as a function of applied pressure (mean ± SD, N = 3). h) Current responses to 5 mV square waves with PEDOT/Pt‐Ir electrodes in comparison with stainless steel electrodes. i) In vivo setup for piezoionic nerve stimulation using PEDOT/Pt‐Ir electrodes, with simultaneous EMG signal recording during stimulation. (a‐b) Reproduced with permission.^[^ ^81^ ^]^ Copyright 2024, Wiley‐VCH. (c‐e) Reproduced with permission.^[^ ^88^ ^]^ Copyright 2021, Elsevier. (f‐i) Reproduced with permission.^[^ ^1^ ^]^ Copyright 2022, American Association for the Advancement of Science.

To address the limitations associated with faradaic reactions at the electrode–gel interface, recent efforts have focused on the development of ion‐conducting gels engineered to operate stably over a broad electrochemical window (ECW). One effective strategy involves incorporating salts or ionic liquids into the gel matrix to reduce the activity of water, thereby expanding the otherwise narrow electrochemical stability window of aqueous systems.^[^ ^79^, ^80^, ^81^, ^82^, ^83^ ^]^ For example, a study utilizing a Sodium alginate/Polyacrylamide (SA/PAAm)‐based hydrogel doped with an organic solvent (DMF) and a salt (NaClO_4_) demonstrated significant suppression of water activity, resulting in a uniform ionic distribution within the polymer network and a broadened ECW (**Figure** **3**a).^[^ ^84^ ^]^ In particular, ClO_4_^−^, a chaotropic anion with high oxidative stability, was shown to inhibit undesirable adsorption at the electrode–electrolyte interface, thus elevating the onset potential for Faradaic reactions and expanding the ECW from ≈2.0 V to over 3.5 V (Figure 3b). These findings highlight the critical role of embedded ions in enabling stable device operation even under high‐voltage conditions.

Beyond enhancing electrochemical stability, ion‐based systems also enable non‐Faradaic charge injection mechanisms.^[^ ^43^, ^85^, ^86^ ^]^ Upon application of external potential, mobile ions within the gel migrate and form an electric double layer (EDL) at the electrode interface, facilitating charge transfer without redox reactions. This process minimizes chemical damage to interfacing biological tissues and allows for safer and more sustainable electrical stimulation.^[^ ^87^, ^88^, ^89^ ^]^ Such principles have been effectively employed in studies aiming to accelerate wound healing using ion‐conducting gel‐based stimulation platforms, underscoring their potential in therapeutic bioelectronic applications. In this system, mechanical energy harvested by a triboelectric nanogenerator (TENG) drives directional ion migration within the gel matrix, resulting in the formation of an electric double layer (EDL) at the gel–tissue interface.^[^ ^90^ ^]^ This EDL enables non‐Faradaic, capacitive charge injection into surrounding biological tissues without inducing electrochemical reactions, such as redox activity or electrode degradation (Figure 3c–d). In vivo studies using a murine wound model demonstrated significantly accelerated healing in the Gel–TENG‐treated group compared to controls (Figure 3e). These findings indicate that the TENG‐induced electrical stimulation was effectively delivered to the wound site via EDL‐mediated ionic charge transfer, without triggering inflammatory responses or causing electrode corrosion—thereby validating the therapeutic potential and biocompatibility of the system.

Furthermore, extensive research has been dedicated to the development of ion‐based self‐powered devices that enable stable charge injection while mitigating the complications associated with external power supplies in vivo.^[^ ^91^, ^92^ ^]^ To enhance the power output of such devices, strategies have focused on modulating the ion distribution and mobility within ion‐conducting gels in response to mechanical stimuli, such as pressure (Figure 3f).^[^ ^1^ ^]^ Under compressive forces, cations and anions—due to their inherent differences in size and mobility—migrate asymmetrically within the gel, resulting in directional charge separation and the generation of an internal electric field (Figure 3g).^[^ ^93^ ^]^ This pressure‐induced ionic polarization enables the gel to produce output voltages that scale linearly with applied force, as demonstrated across various ion species (Figure 3h). Notably, the generated voltage reached levels sufficient to stimulate biological tissues without the need for external power sources. In vivo experiments involving neural stimulation in a murine model confirmed the functional efficacy of this piezoionic mechanism, where self‐generated electrical signals elicited clear electromyographic (EMG) responses, validating the feasibility of autonomous, bio‐integrated stimulation (Figure 3i). The electrochemical stability of ion‐conducting gels—achieved by suppressing redox reactions, enabling non‐Faradaic and self‐powered charge injection via mobile ions, and minimizing the reliance on external power sources. As a result, ensuring electrochemical stability has become a fundamental requirement for the safe and reliable operation of implantable bioelectronic systems.

### 2.2 Ionic Retention and Leaching Stability

Maintaining a stable ionic environment within ion‐conducting gels is essential for the reliable performance of bioelectronic devices, as uncontrolled ion loss can lead to diminished conductivity,^[^ ^94^ ^]^ compromised signal fidelity,^[^ ^95^ ^]^ and increased risk of adverse electrochemical reactions.^[^ ^96^ ^]^ Effective control of ion retention and minimization of ion leaching are therefore fundamental to ensuring long‐term device stability and biocompatibility under physiological conditions. To address these challenges, recent advances have focused on strategies such as tuning ion mobility via polymer chain engineering,^[^ ^97^ ^]^ implementing barriers to prevent ion diffusion across the gel interface,^[^ ^98^ ^]^ and leveraging endogenous biological ions to suppress unwanted side reactions.^[^ ^99^ ^]^ In the following sections, we examine these approaches in detail, highlighting their roles in enhancing the functional longevity of ion‐conducting gels for bioelectronic applications.

**Figure 4:**
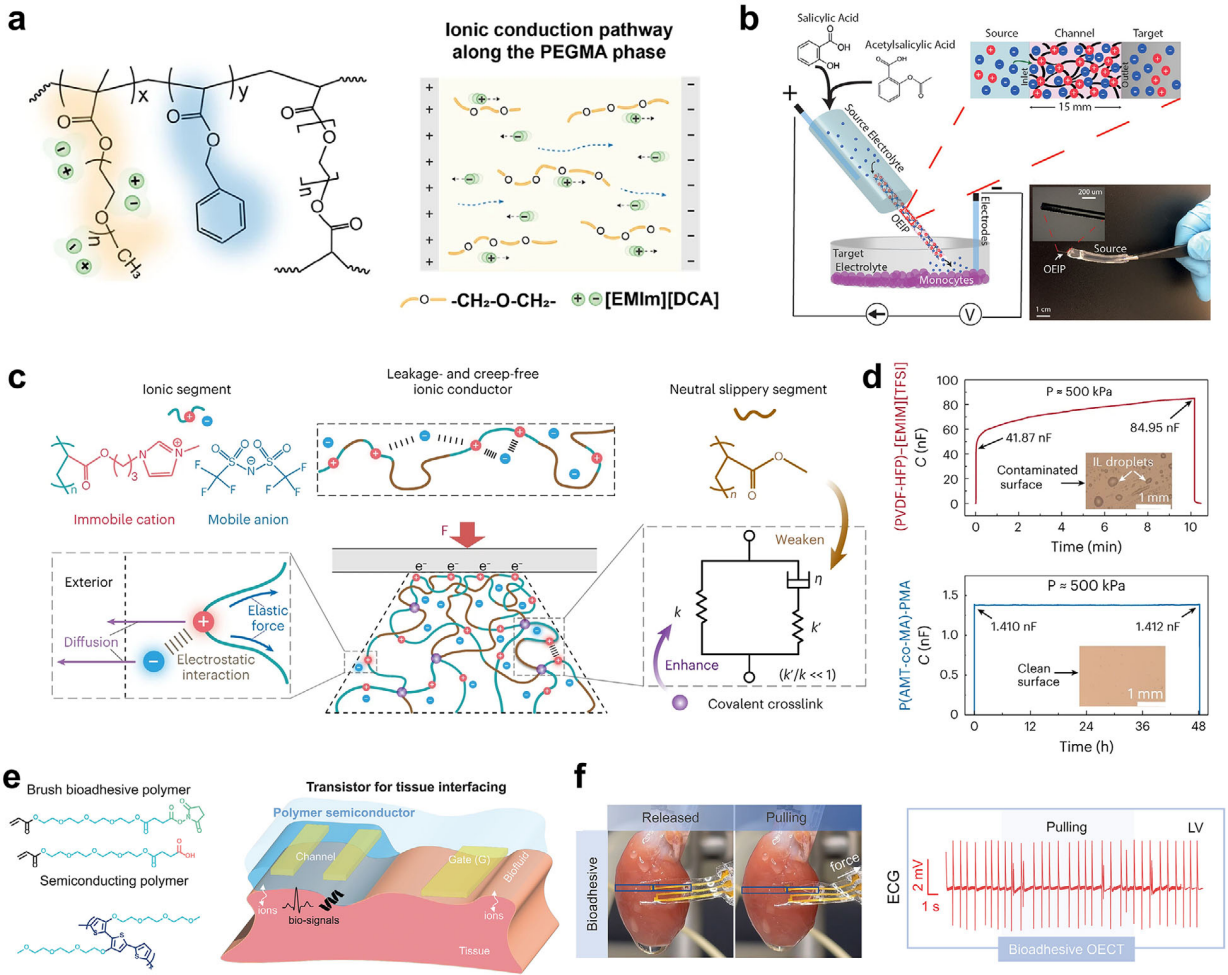
a) Chemical structure of the crosslinked nanostructured ionogel and schematic presentation of ionic pathway along the PEGMA phase. b) Schematic illustration of an implantable drug‐delivery system based on an organic electronic ion pump (OEIP), which enables ion‐selective transport through a polyelectrolyte channel. c) Schematic illustration of the design principles for a leakage‐free and creep‐resistant polyelectrolyte elastomer, enabling stable, drift‐free performance in iontronic sensing applications. d) Comparison of capacitance stability and electrode condition between PEE‐based and ionogel‐based sensors under static compression. e) Chemical structure and device schematic of bioadhesive polymer semiconductors designed for electrochemical transistor‐based tissue interfacing. f) Schematic and functional demonstration of a bioadhesive semiconductor‐based electrochemical transistor operated by bio‐ions for stable ECG signal recording. (a) Reproduced with permission.^[^ ^98^ ^]^ Copyright 2024, Springer Nature. (b) Reproduced with permission.^[^ ^99^ ^]^ Copyright 2019, Wiley‐VCH. (c‐d) Reproduced with permission.^[^ ^101^ ^]^ Copyright 2024, Springer Nature. (e‐f) Reproduced with permission.^[^ ^102^ ^]^ Copyright 2023, American Association for the Advancement of Science.

Enhancing the ionic conductivity of ion‐conducting gel‐based bioelectronic devices is crucial for reducing power consumption and achieving rapid response times, thereby enabling precise and high‐performance operation. Recent advances have focused on controlling ion mobility through polymer chain engineering strategies, which tailor the polymer architecture to optimize ionic transport properties within the gel matrix. **Figure** **4**a illustrates a stretchable ionogel synthesized through photopolymerization‐induced microphase separation of a block copolymer consisting of poly(ethylene oxide) (PEO) and poly(methyl methacrylate) (PMMA) segments. The PEO blocks, rich in ethylene oxide (EO) chains, impart hydrophilicity and form strong hydrogen bonds with water, creating continuous ion‐conductive pathways that enhance ionic mobility and conductivity.^[^ ^100^ ^]^ Meanwhile, the PMMA blocks provide mechanical strength and phase separation, establishing a stable nanoscale morphology that supports efficient ion transport while maintaining mechanical robustness. This deliberate block copolymer design enables improved ionic conductivity and reduced ion leaching, making the ionogel well‐suited for durable and high‐performance bioelectronic applications. Additionally, an implantable drug delivery system based on an organic electronic ion pump has been developed, enabling ion‐selective transport through the use of polyelectrolytes (Figure 4b). By employing polymer chain engineering to regulate ion mobility,^[^ ^101^ ^]^ this approach successfully fabricates ion‐controlling materials capable of releasing specific target molecules with high precision.

However, ion mobility can cause ion leaching from ion‐conducting gels, leading to uneven ionic distribution and reduced sensing accuracy.^[^ ^102^ ^]^ This undermines device reliability, especially for long‐term or implantable applications. Therefore, minimizing ion leaching is crucial to maintain stable ionic environments, ensuring consistent performance and accurate biosignal detection. To address the challenge of ion leaching and its impact on sensor reliability, recent advances have focused on developing leakage‐free and creep‐resistant ion‐conducting gel.^[^ ^103^ ^]^ The common issue of signal drift in conventional iontronic sensors, which arises from ionic conductor creep and component leakage, occurs under sustained mechanical loading. To overcome this, design principles for polyelectrolyte elastomers have been proposed to eliminate leakage and resist creep, thereby enabling stable and drift‐free sensor performance as schematically shown in Figure 4c. Figure 4d contrasts capacitance stability and electrode integrity between sensors based on polyelectrolyte elastomers (PEE) and traditional ionogels under static compression. The PEE‐based sensors exhibit superior capacitance retention and maintain electrode condition, resulting in enhanced sensing accuracy relative to conventional sensors. Quantitative metrics such as drift ratio and drift rate are defined to systematically evaluate signal stability. These findings underscore the importance of material design in minimizing ion leakage and mechanical creep to achieve reliable, high‐fidelity bioelectronic sensing.

Another effective strategy to minimize ion leaching and mitigate adverse side reactions involves leveraging endogenous biological ions rather than relying solely on exogenous ionic components. This approach enhances biocompatibility and reduces potential toxicity by utilizing the native ionic environment within the body. Recent developments include bioadhesive polymer semiconductors specifically designed for electrochemical transistor‐based tissue interfaces, as depicted in Figure 4e.^[^ ^104^ ^]^ These materials harness bio‐ions to enable stable device operation without the need for added ionic species. Figure 4f demonstrates the practical application of such bioadhesive semiconductor electrochemical transistors, which achieve consistent and high‐fidelity ECG signal recording by directly interfacing with physiological ions. This paradigm highlights a promising direction for next‐generation bioelectronic systems that integrate seamlessly with the body's own ionic milieu to maintain long‐term stability and functionality. Collectively, these advancements in ion retention and ionic regulation within ion‐conducting gels are essential for creating bioelectronic devices that achieve high sensitivity, stability, and biocompatibility required for long‐term in vivo functionality.

### 2.3 Mechanical Stability

Ensuring robust mechanical integrity in ion‐conducting gels is essential for their effective performance in bioelectronic devices, which are subject to repetitive deformation and mechanical stress in physiological settings. Dynamic ionic crosslinking, including the use of zwitterionic polymers, creates reversible and adaptable networks that enhance both toughness and elasticity,^[^ ^105^ ^]^ and can be synergistically combined with micro‐/mesoscale architectural cues such as micro‐pyramid texturing in double‐network ionic organohydrogels to concentrate benign deformation pathways and increase effective contact area.^[^ ^106^ ^]^ Enhancing these ionic crosslinks through ion‐exchange reactions further strengthens the gel structure, yielding materials with superior mechanical resilience. Moreover, ion–dipole interactions enable intrinsic self‐healing and improved moisture retention, contributing to sustained mechanical robustness and device longevity under demanding biological conditions,^[^ ^107^, ^108^ ^]^ while simple salt treatments such as LiBr immersion establish persistent hydration shells that impart anti‐freezing/anti‐dehydrating stability without sacrificing sensitivity.

Enhancing the mechanical properties of ion‐conducting gels requires the establishment of strong interactions between polymer chains within the network. Recent studies have increasingly focused on the role of ionic liquids incorporated into these systems, demonstrating their significant contribution to reinforcing mechanical robustness; critically, ionic liquids and inorganic salts act as cohesive network modifiers, not merely conductive media, driving energy dissipation via reversible ionic bonds, mitigating fatigue, and stabilizing mechanics in humid or thermally variable environments.^[^ ^109^ ^]^ By facilitating stronger intermolecular interactions and promoting network cohesion, ionic liquids serve not only as conductive media but also as integral components that improve the structural integrity and durability of ion‐conducting materials; in parallel, solvent‐ and surface‐engineering steps such as LiBr immersion, polyol–water exchange, and elastomeric or double‐hydrophobic coatings lower water activity and stabilize hydration shells. These treatments reinforce inter‐ and intrachain interactions, preserving tensile strength and delaying crack growth under cyclic loading.^[^ ^110^ ^]^

**Figure 5:**
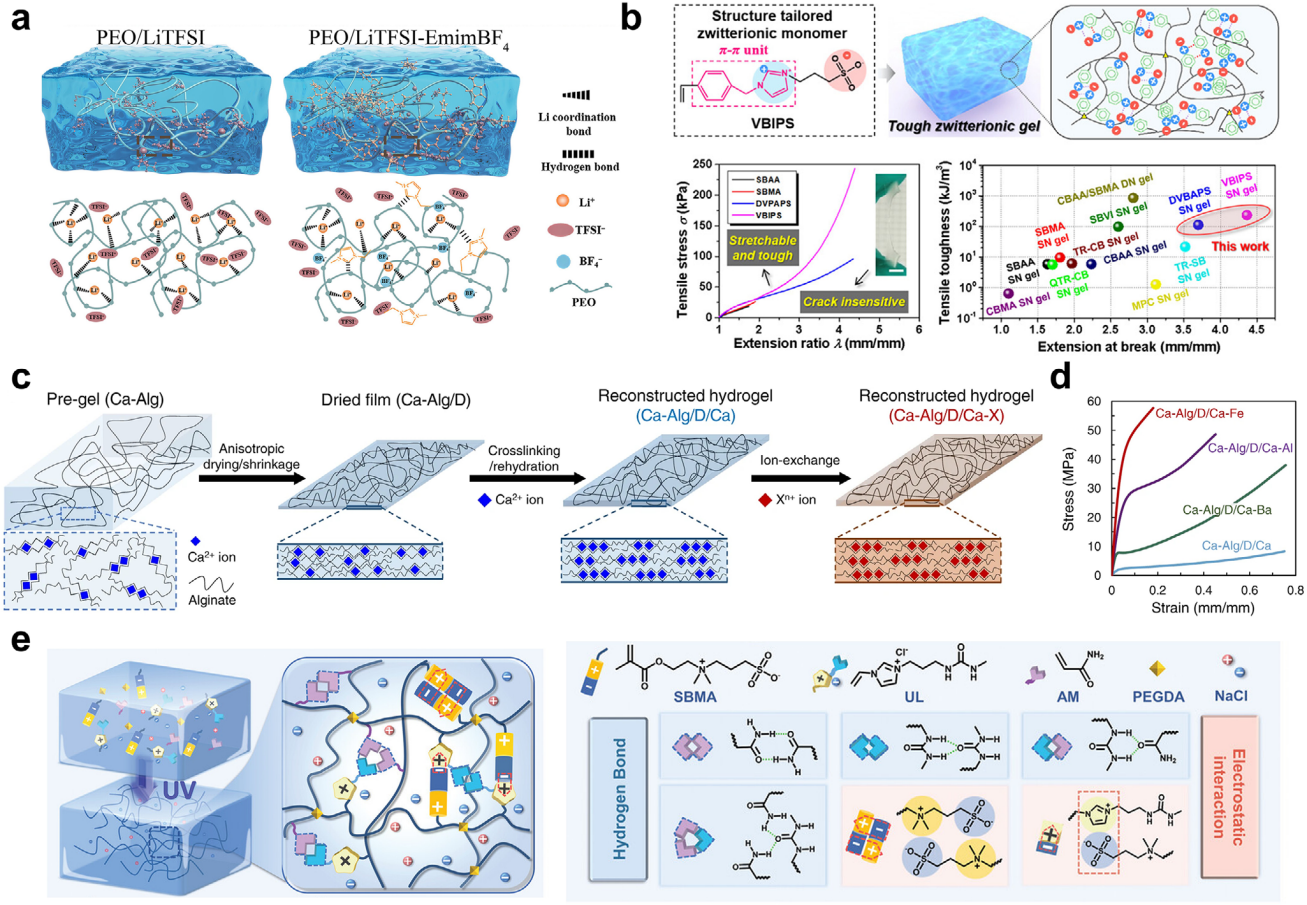
a) Schematic illustration of the ionogel system highlighting the interactions among the polymer (PEO), lithium salt (LiTFSI), and ionic liquid (EmimBF_4_). b) Enhanced mechanical toughness and self‐healing of zwitterionic VBIPS hydrogels via a two‐phase structure, demonstrating flexibility, antifreeze behavior, and antifouling performance for bioelectronic applications. c) Fabrication process of reinforced hydrogels via anisotropic shrinkage, Ca^2^⁺‐induced cross‐linking, and multivalent ion exchange for enhanced mechanical performance. d) Stress‐strain curves of the reconstructed hydrogels. e) Schematic diagram of the preparation of an ionic liquid imidazolium salt with a ureido backbone (UL) based hydrogel. (a) Reproduced with permission.^[^ ^104^ ^]^ Copyright 2023, Association for the Advancement of Science. Springer Nature. (b) Reproduced with permission.^[^ ^105^ ^]^ Copyright 2021, American Chemical Society. (c‐d) Reproduced with permission.^[^ ^106^ ^]^ Copyright 2022, Springer Nature. (e) Reproduced with permission.^[^ ^107^ ^]^ Copyright 2024, American Association for the Advancement of Science.

This study employed the incorporation of reinforcing agents into ionic liquid‐based ionogels to enhance their mechanical properties (**Figure** **5**a).^[^ ^111^ ^]^ The addition of these fillers resulted in a denser and more robust polymer network, effectively addressing the common mechanical weaknesses observed in conventional ionogels. This strengthened network significantly improved the tensile strength and durability of the gels, increasing their resistance to external mechanical stresses and enabling stable long‐term operation. Such an approach offers a promising strategy for designing materials that combine high ionic conductivity with superior mechanical robustness, meeting the demands of bioelectronic applications. Zwitterionic ion‐conducting gels are renowned for their biocompatibility and antifouling properties, but often suffer from limited mechanical strength. In a previous report, the incorporation of a structurally enhanced sulfobetaine monomer containing benzene and imidazole groups significantly improved the mechanical robustness, with the tensile and fracture toughness values increasing by ≈40 and 60 times compared with conventional sulfobetaine‐based zwitterionic hydrogels, respectively (Figure 5b).^[^ ^112^ ^]^ This reinforcement arises from a unique two‐phase microstructure and strong molecular associations, which also enable tunable mechanical properties and self‐healing. These findings highlight the critical role of zwitterionic design in significantly enhancing the mechanical durability of ion‐conducting gels for advanced bioelectronic applications. Building upon these material‐level strategies, further enhancement of mechanical integrity has been achieved by directly modulating the ionic crosslinking strength within the gel network (Figure 5c).^[^ ^113^ ^]^ Rather than relying solely on polymer design or filler incorporation, this approach strategically substitutes weaker ionic crosslinkers with multivalent metal ions that establish stronger and more stable electrostatic interactions (Figure 5d). This ion exchange mechanism provides a more controllable and durable crosslinking environment, leading to markedly improved mechanical strength without sacrificing ionic conductivity.

Another emerging strategy for improving the mechanical reliability and long‐term operability of ion‐conducting gels involves leveraging dynamic ion–dipole interactions within the polymer matrix. Unlike covalent or purely ionic crosslinking mechanisms, ion–dipole interactions provide a balance of reversibility and binding strength, enabling the gel to autonomously repair mechanical damage and retain its hydration state under physiological conditions. Recent developments have demonstrated that incorporating dipole‐rich polymer segments, such as sulfonated polyurethanes or urea‐based motifs, facilitates the formation of reversible bonds with mobile ions and results in dynamic networks that can reorganize after deformation (Figure 5e).^[^ ^114^ ^]^ In this context, the integration of thermodynamically favorable ion–dipole associations not only reinforces the gel structure but also prevents dehydration, a critical factor for preserving ionic conductivity and mechanical flexibility over time. As illustrated in Figure 5c, such a design enables robust, water‐retentive, and self‐healing ion‐conducting gels, offering a promising material foundation for durable and adaptive bioelectronic systems. Quantitative values from the cited studies are harmonized in **Table** **1** to enable like‐for‐like comparison across material classes.

**Table 1:** Summary of mechanical stability of ion‐conducting gels.

Classification	Mechanical property	Ref
Hydrogel	Stretchability: 370%	[84]
	Stretchability: 100%	[104]
	Stretchability: 440%	[112]
	Young's modulus: 1000 MPa	[113]
	Stretchability: 1075%, Young's modulus: 98 kPa	[114]
	Stretchability: 2044%, Young's modulus: 498.3 kPa	[127]
Iongel	Stretchability: 120%	[100]
	Stretchability: 61.3%, Young's modulus: 1120 kPa	[103]
	Stretchability: 100%	[111]
	Stretchability: ≈50%, Young's modulus: 6000 kPa	[128]
Eutectic gel	Stretchability: >600%, Young's modulus: 760–2850 MPa(–20–60 °C)	[126]
Organogel	Stretchability: 260%, Young's modulus: 12 kPa	[90]

In sum, these diverse strategies underscore a multifaceted approach to achieving mechanical stability in ion‐conducting gels. By integrating dynamic ionic crosslinking, structural reinforcement through zwitterionic or dipole‐rich architectures, and targeted ion‐exchange mechanisms, researchers have established design principles that address both strength and adaptability. Such integrative methods allow the formation of gels that not only resist mechanical fatigue but also autonomously recover from damage and maintain functional hydration. These advances are pivotal for developing ion‐conducting materials capable of enduring prolonged mechanical stress and deformation, ultimately ensuring reliable performance in next‐generation bioelectronic platforms.

### 2.4 Thermo‐Physiological Stability

**Figure 6:**
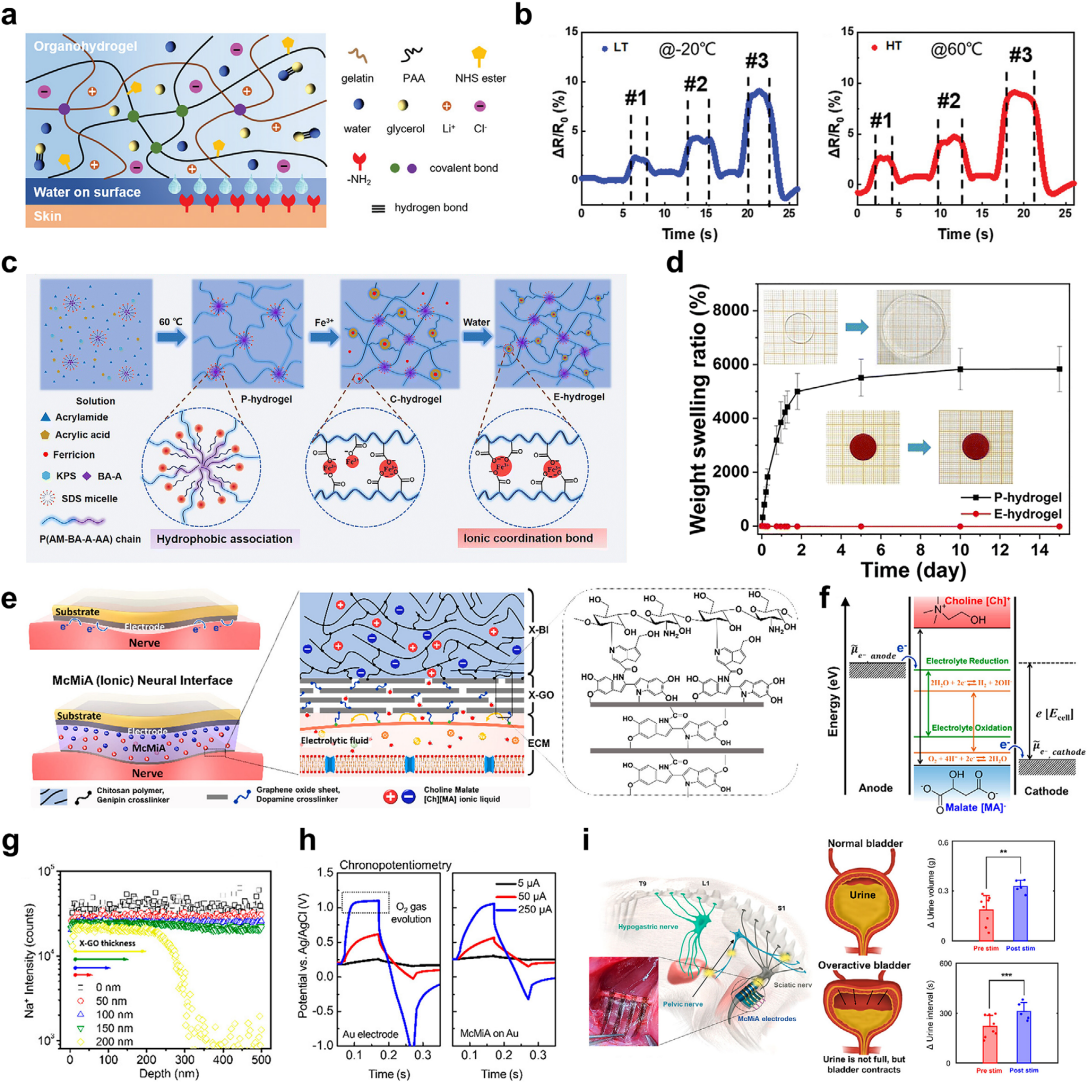
a) Material strategy scheme of gel stable in thermal environments. Thermal compatibility and anti‐dehydration properties were achieved via the addition of glycerol. b) Consistent and stable performance of the ion gel with temperature. c) Strategy of anti‐swelling hydrogel via coordination bonding of Fe3^+^. d) Weight swelling ratio of P and E‐hydrogel in deionized water; the inset shows the swelling of the gel after 15 days. e) Schematic diagram of material strategy for ion‐conducting gels that operates reliably in physiological environments. f) Thermodynamic potential and ECW of [Ch]^+^[MA]^−^ ILs with various water contents. g) ToF‐SIMS depth profiles of Na+ ions from McMiA penetrated in PBS. X‐GO layer thickness above 200 nm sufficiently shows the effect of preventing ion exchange. h) Voltage responses of a commercial Au electrode and the McMiA film on the Au electrode in PBS environment under biphasic current pulses (5, 50, and 250 µA). i) Scheme of the bladder nervous system and analysis of volume and urination interval showing urination activity (bars: mean ± SD). (a,b) Reproduced with permission.^[^ ^118^ ^]^ Copyright 2021, Wiley‐VCH. (c‐d) Reproduced with permission.^[^ ^119^ ^]^ Copyright 2022, Royal Society of Chemistry. (e‐i) Reproduced with permission.^[^ ^120^ ^]^ Copyright 2023, American Chemical Society.

To ensure the reliable performance of ion‐conducting gels across diverse physiological environments, maintaining stable physicochemical properties is essential. Gel instability often arises from temperature‐induced perturbations and ionic fluctuations in surrounding biological media.^[^ ^115^, ^116^ ^]^ Specifically, thermal stimuli can induce volumetric and conductivity changes through solvent phase transitions—such as freezing at sub‐physiological temperatures or evaporation under elevated thermal conditions.^[^ ^117^, ^118^ ^]^ Additionally, dynamic ion exchange with the extracellular matrix (ECM), driven by pH fluctuations in the local microenvironment, can further disrupt ionic conductivity and structural integrity.^[^ ^119^, ^120^ ^]^ Therefore, endowing ion‐conducting gels with anti‐freezing and anti‐evaporation capabilities is critical for preserving their electrochemical and mechanical stability across a broad physiological temperature range. The freezing of ion‐conducting gels primarily arises from specific intermolecular interactions between solvent molecules that promote crystallization at sub‐zero temperatures.^[^ ^121^ ^]^ By disrupting these interactions, the freezing point of the gel can be effectively lowered, enabling stable performance in cold environments. At elevated temperatures, solvent evaporation becomes a significant concern, as solvent molecules diffuse out of the gel and subsequently evaporate. This issue is especially critical in hydrogel systems due to the high vapor pressure of water, which accelerates solvent loss. To ensure thermal stability while retaining the intrinsic mechanical and electrochemical properties of the gel, it is essential to stabilize the internal solvent content. One effective strategy for imparting anti‐freezing functionality involves weakening the interactions among solvent molecules and introducing additives that preferentially form strong interactions with the solvent.^[^ ^122^, ^123^ ^]^ These modifications help suppress crystallization and minimize phase separation, thereby improving the environmental resilience of ion‐conducting gels. Accordingly, rational material design strategies are required to stabilize the internal solvent phase without compromising the inherent mechanical, ionic, and electrochemical properties of ion‐conducting gels. A representative approach is the development of solvent‐free supramolecular ion‐conductive elastomers (SF‐supra‐ICE), where a naturally occurring ionizable compound (inositol hexakisphosphate, IP_6_) is encapsulated by biocompatible PVA chains through high‐density hydrogen bonding.^[^ ^124^ ^]^ This design fundamentally eliminates the dehydration problem of hydrogels while maintaining high ionic conductivity (>3.3 × 10^−2^ S m^−1^), skin‐like softness, and long‐term electrochemical stability under ambient conditions. Thanks to its solvent‐free nature, the SF‐supra‐ICE can sustain stable electrical and mechanical performance for over 150 days in open air, offering a promising alternative to hydrogel‐ or ionogel‐based systems for skin‐interfaced bioelectronics. One effective approach involves reducing the strength of intermolecular interactions between solvent molecules while incorporating additives that establish stronger and more preferential interactions with the solvent.^[^ ^125^ ^]^ This dual mechanism suppresses solvent crystallization and enhances the thermal resilience of the gel network. An effective strategy to impart anti‐freezing and anti‐dehydration properties to ion‐conducting hydrogels was demonstrated by introducing a binary glycerol–water solvent system into a commercially available double‐network hydrogel composed of gelatin and polyacrylic acid (PAA)^[^ ^126^ ^]^ (**Figure** **6**a). Glycerol, a polyhydric alcohol with abundant hydroxyl groups, forms extensive hydrogen bonding with water molecules when incorporated into the hydrogel matrix. These strong glycerol–water interactions disrupt the intrinsic hydrogen bonding network among water molecules, thereby inhibiting ice crystallization under sub‐zero temperatures and functioning as an effective antifreeze agent. Moreover, due to its high affinity for water through hydrogen bonding, glycerol significantly suppresses water evaporation at elevated temperatures. As a result, hydrogel is able to retain its physical and electrical properties even under thermal stress, enhancing its reliability across a broad range of environmental conditions. As shown in Figure 6b, the double‐network, dual‐solvent hydrogel incorporating glycerol exhibited stable output signals across a wide temperature range. The hydrogel maintained excellent mechanical properties, with tensile strengths of 0.62 MPa at 25 °C and 0.76 MPa at −20 °C, as well as high ionic conductivity (≈6 mS cm^−1^). These characteristics enabled the hydrogel to generate distinct and reproducible electrical signals in response to grasping objects of varying shapes and stiffness, demonstrating its potential for reliable sensing performance under diverse environmental and mechanical conditions.

Meanwhile, in physiologically moist environments, water can infiltrate ion‐conducting gels and cause swelling, which alters their mechanical properties. This effect becomes more severe under fluctuating pH conditions, especially in gels with high ion concentrations. Swelling can cause significant changes in volume and stiffness, which can interfere with stable biosignal sensing or precise electrical stimulation. To prevent this, anti‐swelling functionality is needed to maintain consistent mechanical performance and ensure reliability under physiological conditions. Recently, this issue has been effectively addressed by introducing strong crosslinking within conductive hydrogels^[^ ^127^ ^]^ (Figure 6c). A polymer backbone rich in carboxyl groups was coordinated with iron ions to form a stable gel network. Upon rinsing with water to remove excess iron, the mixed coordination states of iron evolved into predominantly trivalent coordination, resulting in more robust crosslinking points. The resulting ion‐conducting gels, termed equilibrium swollen hydrogels (E‐hydrogels), exhibited enhanced mechanical strength due to dense chemical crosslinking. Increasing the number of coordination bonds led to higher rigidity but reduced deformability. The optimized E‐hydrogel demonstrated outstanding mechanical performance, with a tensile strength of ≈4.52 MPa, modulus of 498.3 kPa, elongation at break of ≈2044%, and toughness of 58.415 MJ m^−3^. As shown in Figure 6d, the strong coordination crosslinks effectively endowed the ion‐conducting hydrogel with anti‐swelling properties. While the uncrosslinked and unwashed control sample exhibited a swelling ratio of up to 5500%, the engineered E‐hydrogel showed only ≈9% volume change over 10 days, attributed to its dense network structure and robust coordination bonding. Furthermore, the E‐hydrogel maintained excellent dimensional stability across various solvent environments, exhibiting strong swelling resistance over a wide pH range (pH 2–11) for up to 15 days. Additional experiments confirmed that hydrogel preserved its mechanical integrity under these diverse pH conditions. These results demonstrate a promising strategy to enhance the reliability of output signals from ion‐conducting gels by ensuring consistent performance under physiologically and chemically dynamic environments.

Finally, to ensure stable operation of ion‐conducting gels under physiological conditions, it is critical to minimize mass transfer between the gel and the surrounding extracellular matrix (ECM). Leakage of internal ions or solvents may not only pose potential cytotoxicity but also disrupt local ionic homeostasis, leading to long‐term cellular damage. Given the inherently porous nature of hydrogels, such risks must be carefully considered. Therefore, strategies that establish a well‐defined interface to isolate the gel from biological tissues are essential for long‐term biocompatibility and functional reliability. One effective approach involves the construction of a multicross‐linked membrane–ionogel assembly (McMiA) that functions as an ion‐selective barrier at the gel–tissue interface^[^ ^128^ ^]^ (Figure 6e). This system integrates a dopamine‐cross‐linked graphene oxide membrane with a biopolymer‐based ionogel to prevent undesired ion exchange with surrounding biological media. As illustrated in Figure 6f, the graphene oxide layer is positioned between the ionogel and the extracellular matrix and, with a thickness exceeding 200 nm, effectively restricts ion diffusion. After 24 h of immersion in PBS, sodium ions were detected only outside the gel, confirming the barrier function of the graphene oxide layer. Furthermore, the addition of Choline‐Malate Ionic liquids broadened the electrochemical window (ECW) and minimized side faradaic current, preventing undesirable gas evolution at the neural interface (Figure 6g). As shown in Figure 6h, the Au electrode exhibited a plateau region corresponding to the oxidation‐reduction reaction, whereas McMiA did not show such behavior in the chronopotentiometry graph, demonstrating its potential for stable neurostimulation. Finally, McMiA was applied to sciatic nerve stimulation for therapeutic improvement in a rat model of interstitial cystitis (Figure 6i). McMiA showed an increase in urine volume and a decrease in urine interval, demonstrating its potential as a promising platform for advanced neural interface engineering. These approaches collectively establish a materials framework that ensures mechanical robustness, ionic stability, and biocompatibility of ion‐conducting gels under thermal and physiological conditions, thereby enabling reliable operation in bioelectronic applications.

This section consolidates design strategies that underpin sustainability at the materials level: i) electrochemical stability through electrochemical‐window (ECW) expansion and ii) ion‐leaching suppression; iii) mechanical durability via dynamic ionic crosslinking, zwitterionic/dipole‐rich architectures, and ion‐exchange strengthening; and iv) thermo‐physiological stability through anti‐freezing/anti‐dehydration measures in physiological media. Together, these guidelines link composition, network structure, and ionic transport, providing material‐level rules that directly support reliable biosignal transduction and safe stimulation in downstream devices.

## 3 Device Sustainability of Ion‐Conducting Gels for Bioelectronic

Building on these material‐level rules for ECW, ion‐leaching, mechanical robustness, and thermo‐physiological stability, this section examines device architectures that optimize ion–electron coupling, minimize power at low voltage, and preserve performance under physiological stress. While the properties of ion‐conducting gels are critical to enabling bioelectronic function, their integration into devices requires careful consideration of how these materials perform under physiological constraints. Ensuring stability at the device level is thus equally important for realizing their full potential in practical applications.

### 3.1 Biocompatibility

Achieving long‐term operational stability under physiological conditions in ion‐conducting gel–based implantable devices requires not only material durability but also stable and biocompatible interactions with surrounding tissues and cells. Insufficient biocompatibility may elicit inflammatory responses,^[^ ^129^ ^]^ resulting in tissue damage^[^ ^130^ ^]^ and compromised device function,^[^ ^131^ ^]^ and in some cases, requiring surgical intervention. Conventional ion‐conducting gels often exhibit limited antimicrobial activity, while chemically synthesized variants may induce cytotoxic effects.^[^ ^132^ ^]^

Accordingly, antifouling and antibacterial interfaces that preserve surface hydration and deter nonspecific protein or cell adhesion can mitigate foreign‐body responses and help maintain device function.^[^ ^133^, ^134^ ^]^ In parallel, biocompatible hydrogel designs that prioritize anti‐inflammatory, pro‐healing, and tissue‐integrative responses support long‐term compatibility at cellular and organ levels.^[^ ^133^, ^135^ ^]^

Beyond these concerns, it is also essential to recognize potential toxicological risks associated with the common constituents of ion‐conducting gels. Ionic liquids, frequently employed to enhance ionic conductivity and broaden electrochemical stability windows, can display dose‐dependent cytotoxicity by disrupting cellular membranes or mitochondrial activity during prolonged exposure.^[^ ^136^ ^]^ Similarly, crosslinkers such as glutaraldehyde, carbodiimide derivatives, or diazirine‐based reagents, while effective in stabilizing gel networks, may leave reactive byproducts that provoke local inflammation or even genotoxic effects if not fully removed.^[^ ^137^ ^]^ Metal ions (e.g., Ca^2^⁺, Fe^3^⁺, Zn^2^⁺), though often used to reinforce gel structures or provide bioactivity, can catalyze the generation of reactive oxygen species (ROS) and cause oxidative stress, thereby disturbing cellular homeostasis under chronic implantation conditions.^[^ ^138^ ^]^ Even widely adopted polymeric backbones such as PEDOT:PSS, polyacrylamide, or poly(vinyl alcohol) may degrade into smaller fragments capable of eliciting inflammatory responses or altering the local ionic balance.^[^ ^139^ ^]^ Additionally, uncontrolled ion leaching or solvent leakage from gels can destabilize the extracellular ionic environment, impair electrophysiological function, and raise the risk of systemic toxicity.^[^ ^140^ ^]^

This section reviews recent advances in enhancing biocompatibility by integrating antimicrobial functionalities into ion‐conducting gel systems.

**Figure 7:**
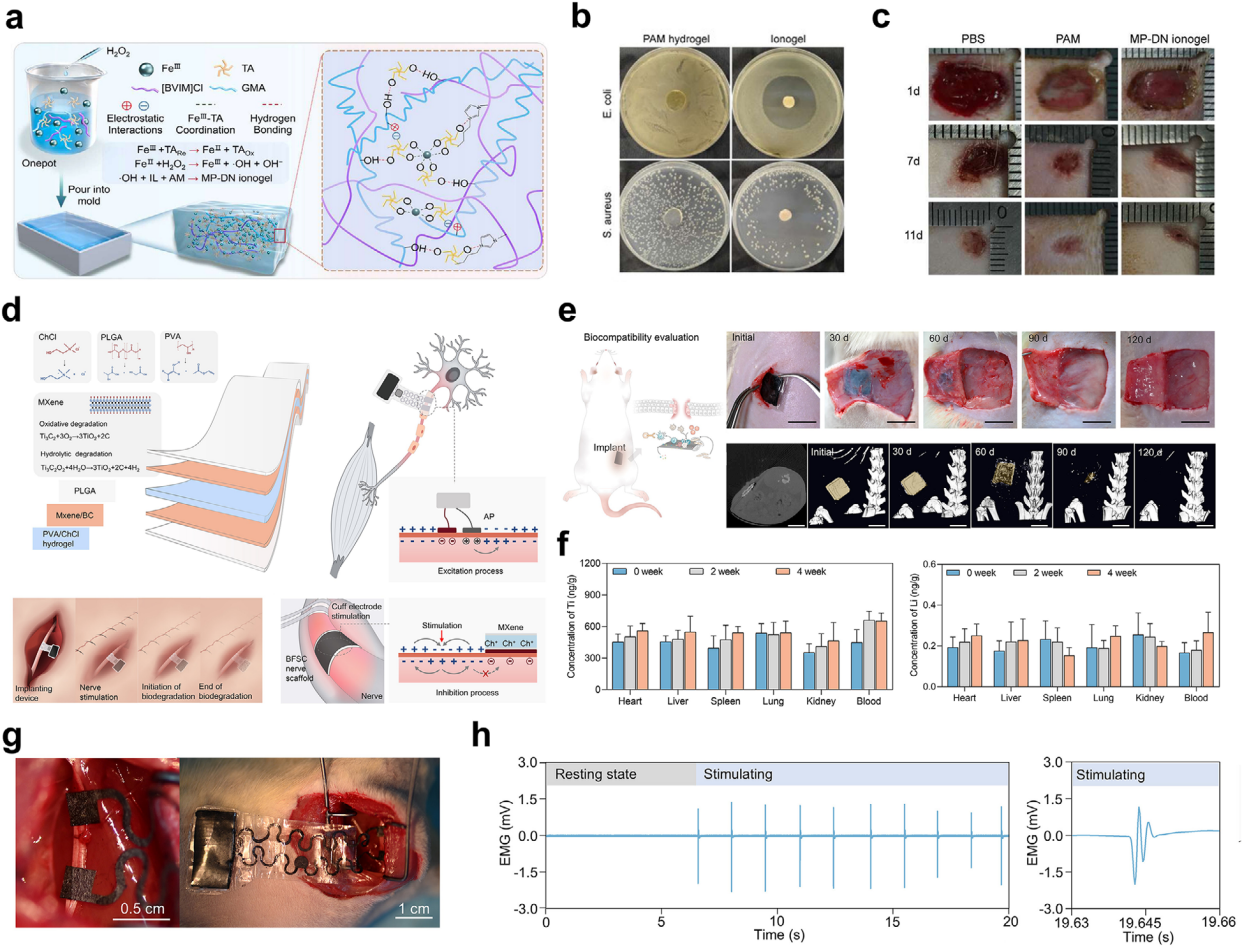
a) Schematic illustration of the synthesis and properties of MP‐DN ionogel. b) Images of antibacterial zones against E. coli and S. aureus using control hydrogel and the MP‐DN ionogel. c) Images showing the wound‐healing process following different treatments of infected wounds. d) Schematic illustration of the structure, components, and in vivo degradation of the supercapacitor. e) Optical photographs and CT images of in vivo degradation of BFSCs implanted subcutaneously in rats. f) In vivo distribution of Ti and Li elements in major organs after BFSC implantation, measured by ICP‐MS. g) Image of the BFSC bioresorbable electrical stimulation system. h) EMG signals recorded during stimulation. (a‐c) Reproduced with permission.^[^ ^125^ ^]^ Copyright 2025, Elsevier. (d‐h) Reproduced with permission.^[^ ^128^ ^]^ Copyright 2025, Elsevier.

Incorporating metal ions into ion‐conducting gel offers an effective strategy to simultaneously stabilize the gel network and impart antibacterial functionality.^[^ ^141^ ^]^ The metal–phenolic dual‐network ionogel (MP‐DN ionogel) was synthesized using Fe^3^⁺–tannic acid and H_2_O_2_ as a dual self‐catalyzing system to initiate the copolymerization of a hydrophilic ionic liquid monomer and a hydrophobic acrylamide glycidyl ester monomer(**Figure** **7**a). The resulting metal–phenolic network (MPN), an organic–inorganic hybrid structure, imparts anti‐inflammatory, antioxidant, and the use of this antibacterial ion‐conducting gel resulted in cell viability comparable to the control, confirming the fabrication of a highly biocompatible ion‐conducting gel. The antibacterial activity of the ion‐conducting gel was evaluated by comparing the inhibition of *E. coli* (Gram‐negative) and *S. aureus* (Gram‐positive) growth (Figure 7b).^[^ ^142^ ^]^ While the Polyacrylamide (PAM) hydrogel exhibited no observable inhibition zone, the MP‐DN ionogel formed a pronounced antibacterial zone, indicating its potential to prevent inflammation and maintain biocompatibility in physiological environments. This ion‐conducting gel was applied as a wound healing patch in a full‐thickness tissue injury model, demonstrating its effectiveness in promoting tissue regeneration (Figure 7c).

In addition to ensuring the biocompatibility of ion‐conducting hydrogels, various material properties may be required for their application as implantable devices. One strategy that can be employed is the introduction of natural degradable characteristics, which would eliminate the need for additional surgery to remove the device after its use is completed.^[^ ^143^ ^]^ Building on this strategy, recent studies have focused on constructing fully biodegradable iontronic devices by integrating biocompatible and bioresorbable materials into each component.^[^ ^144^ ^]^ For instance, a biodegradable and flexible supercapacitor (BFSC) was developed using MXene/bacterial cellulose (BC) composite electrodes, a PVA/choline chloride‐based hydrogel electrolyte, and a Poly(lactic‐co‐glycolic acid) (PLGA) encapsulation layer (Figure 7d). The use of choline chloride, a biocompatible ionic salt, imparted high ionic conductivity and enhanced flexibility through hydration‐driven swelling of the hydrogel matrix. Importantly, all components were designed to undergo complete degradation under physiological conditions via chemical, enzymatic, or physical mechanisms, eliminating the need for surgical retrieval. The in vivo biodegradation of the BFSC was validated through subcutaneous implantation in rats, where hydrogel decomposition was triggered by physiological water and enzymatic infiltration. Optical imaging and CT scans confirmed the complete, nontoxic degradation of the device into TiO_2_ over a 120‐day period (Figure 7e). Furthermore, the biosafety of the degradation process was demonstrated by monitoring Ti and Li accumulation in major organs at two‐week intervals, which revealed no significant elemental retention or tissue toxicity throughout the degradation period (Figure 7f). Controlled electrical stimulation of the sciatic nerve using the integrated BFSC system effectively modulated EMG signals in the hindlimb, resulting in stable neuromodulatory inhibition (Figures 7g–h). The system, comprising a biodegradable supercapacitor for power, a pressure‐activated switch, stimulation electrodes, and a current transmission circuit, elicited reliable hindlimb actuation upon implantation in a murine model. These advances collectively underscore the critical importance of integrating biocompatibility, antibacterial functionality, and programmed biodegradability into ion‐conducting gel systems to enable safe, effective, and self‐resolving operation of implantable bioelectronic devices under physiological conditions.^[^ ^142^ ^]^

### 3.2 Interface Stability: Adhesion and Conformability Aspects

In bioelectronic systems, the interface between the device and biological tissue plays a crucial role in ensuring signal fidelity, device durability, and biocompatibility.^[^ ^92^ ^]^ Poor interfacial adhesion or mechanical mismatch can cause unstable contact, resulting in motion‐induced artifacts,^[^ ^145^ ^]^ low signal‐to‐noise ratios,^[^ ^146^ ^]^ and eventual device malfunction.^[^ ^147^ ^]^ To overcome these limitations, recent research has focused on the design of ion‐conducting gels with improved interfacial adhesion and mechanical adaptability.^[^ ^148^, ^149^ ^]^ These include printable and deformable gel systems that conform to complex tissue surfaces, as well as biomimetic materials engineered to replicate the mechanical and ionic characteristics of human skin.^[^ ^150^ ^]^ This section discusses recent strategies to enhance interfacial stability, focusing on adhesion reinforcement, formability, and motion‐resilient signal performance for seamless integration in dynamic biological environments.

**Figure 8:**
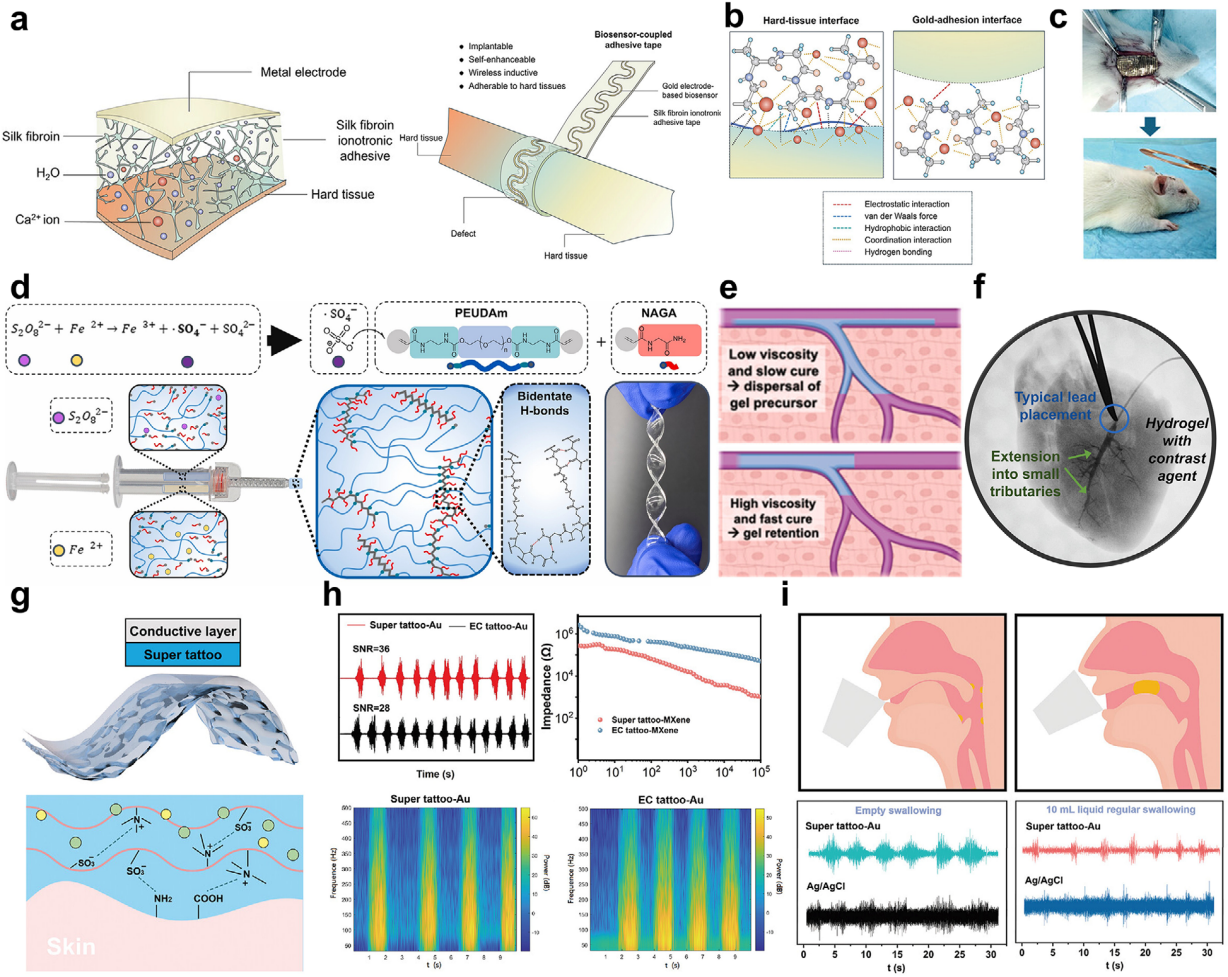
a) Schematic illustration of the structure of the IGBI‐based bioelectronic devices and b) adhesive mechanism. c) Application of the EDC‐IGBI for in vivo wireless monitoring of physiological signals. d) Schematic illustration of the injectable ionic hydrogel electrode system, showing double‐barrel syringe‐mediated redox initiation. e) Schematic illustration of ionic hydrogel distribution in blood vessels as a function of viscosity and in situ gelation behavior. f) Delivery of the ionically conductive hydrogel using a double‐barrel syringe into small tributaries. g) Schematic illustration of super tattoo‐based electrodes for electrophysiology. h) EMG signals and corresponding time‐frequency maps collected using super tattoo‐Au and EC tattoo‐Au electrodes. i) Schematic diagram of muscle activity involved in swallowing. (a‐d) Reproduced with permission.^[^ ^134^ ^]^ Copyright 2025, Wiley‐VCH. (e‐g) Reproduced with permission.^[^ ^137^ ^]^ Copyright 2025, Elsevier. (h‐i) Reproduced with permission.^[^ ^141^ ^]^ Copyright 2024, Wiley‐VCH.

To achieve stable adhesion at biological interfaces, a self‐reinforcing ionogel bioadhesive interface (IGBI) was developed (**Figure** **8**a).^[^ ^151^ ^]^ Silk fibroin was dissolved in a formic acid and CaCl_2_ solvent system, cast into a film, and processed to remove residual acid, resulting in a transparent, stretchable, and adhesive substrate. The resulting IGBI was integrated with a bioelectronic device composed of gold electrodes patterned via mask‐assisted sputtering. The ionogel, composed of biocompatible silk fibroin and calcium ions, exhibited strong tissue adhesion through multiple noncovalent interactions (Figure 8b). Specifically, hydrogen bonding occurred between hydrophilic amino acids in fibroin and hydroxyl or amine groups on tissue surfaces, while hydrophobic domains formed complementary hydrophobic interactions. Additionally, Ca^2^⁺ ions in the ionogel established electrostatic attraction with negatively charged tissue components. This multivalent adhesion mechanism provided enhanced interfacial stability and mechanical integrity, outperforming conventional soft‐tissue adhesives.^[^ ^152^ ^]^ The IGBI demonstrated significantly higher adhesion strength than commercial adhesives across diverse substrates, including bone and metal. When integrated into a wireless bioelectronic device (EDC‐IGBI), it enabled real‐time monitoring of healing at defect sites while promoting osteogenesis via Ca^2^⁺ release (Figure 8c), highlighting its potential for durable tissue integration and orthopedic applications.

Conventional metal electrodes face limitations in reaching deep tissues and may cause mechanical mismatch or side effects. Therefore, it is important to precisely adhere bioelectronic devices to biological interfaces to minimize mechanical gaps and ensure close contact, which stabilizes the interface through mechanical integrity.^[^ ^153^ ^]^ Recently, soft gel‐based electrodes have been developed to enable long‐term, painless electrical stimulation deep within the body, offering a promising strategy for treating arrhythmias without discomfort.^[^ ^154^ ^]^

Following this strategy, PEG was end‐functionalized with carbodiimidazole (CDI) to yield PEG‐CDI, which was subsequently reacted with ethylenediamine to produce PEG‐diamine.^[^ ^155^ ^]^ The terminal amine groups were further modified with acryloyl chloride to form polyether urethane diacrylamide (PEUDAm), a polymer backbone bearing acrylamide moieties. For in situ gelation, ammonium persulfate (APS) and iron gluconate were employed as oxidizing and reducing agents, respectively. Their redox interaction generated sulfate‐based radicals that initiated rapid radical polymerization between the acrylamide groups on PEUDAm and vinyl groups on N‐acryloyl glycinamide (NAGA), forming a 3D ion‐conducting network (Figure 8d). Using a double‐barrel syringe, precursor solutions containing PEUDAm, NAGA, and ionic salts (Na⁺, Cl^−^) were co‐injected along with APS and iron gluconate, enabling fast gelation under physiological conditions without external stimuli. The resulting ion‐conducting gel formed a soft, stretchable network with strong hydrogen bonding, ensuring long‐term interfacial stability with biological tissues.^[^ ^148^ ^]^

Optimal gelation conditions for stable interface formation were investigated through rheological measurements and flow simulations (Figure 8e). While solution viscosity had a limited effect on mixing quality in double‐barrel injection, it significantly influenced gel retention and catheter clogging. In vivo studies in porcine hearts showed that the hydrogel‐filled cardiac veins enabled broad, synchronized stimulation, achieving natural heart contraction, unlike localized metal electrodes (Figure 8f).

In bioelectronic applications, accurate sensing of subtle electrical signals such as EMG and ECG or effective delivery of neural stimulation requires conformal contact and mechanical compatibility with the skin.^[^ ^156^ ^]^ In regions with significant deformation, such as the face or gastrointestinal tract, poor adhesion or mechanical mismatch can lead to device detachment or micro‐motion, causing motion artifacts that distort signal integrity, increase diagnostic errors, and reduce the efficacy of rehabilitation monitoring.^[^ ^157^ ^]^ To address this challenge, it is essential to develop ion‐conducting gels that closely replicate the mechanical and interfacial properties of human skin.^[^ ^158^ ^]^ An ultrathin electronic tattoo, termed “super tattoo,” was developed by integrating a zwitterionic Poly(sulfobetaine methacrylate)–deep eutectic solvent (PSBMA‐DES) gel with electrospun silk fibroin fibers, forming a conformable and adhesive composite structure (Figure 8g).^[^ ^159^ ^]^ The super tattoo electrodes, functionalized with Au, PEDOT:PSS, or MXene, demonstrated significantly lower interfacial impedance and higher signal‐to‐noise ratio compared to conventional ethyl cellulose‐based tattoos. EMG recordings and time‐frequency analyses confirmed superior signal clarity and reduced motion artifacts under dynamic conditions (Figure 8h). The super tattoo was applied to monitor EMG signals during swallowing for dysphagia rehabilitation and demonstrated higher precision and lower motion artifacts compared to conventional Ag/AgCl electrodes, even under movement (Figure 8i).

### 3.3 High‐Performance Device Properties

Ion‐conducting gels offer unique advantages in electrical performance by enabling high sensitivity, stable capacitive charge transfer, and low‐voltage operation.^[^ ^160^, ^161^ ^]^ These properties have been harnessed to develop high‐performance biosensors with precise ionic control, as well as soft stimulators that avoid the tissue damage and electrochemical side effects associated with traditional metal electrodes.^[^ ^162^, ^163^, ^164^, ^165^ ^]^ Additionally, the integration of self‐healing mechanisms ensures long‐term functionality under mechanical stress, further supporting the development of reliable, energy‐efficient ionic devices such as organic electrochemical transistors (OECTs) and ionic diodes.^[^ ^166^, ^167^, ^168^ ^]^ This section highlights recent advancements in enhancing the electrical performance of ion‐conducting gel–based devices, focusing on strategies for improving sensitivity, durability, and energy efficiency in biosensing and neuromodulation applications.^[^ ^169^ ^]^

**Figure 9:**
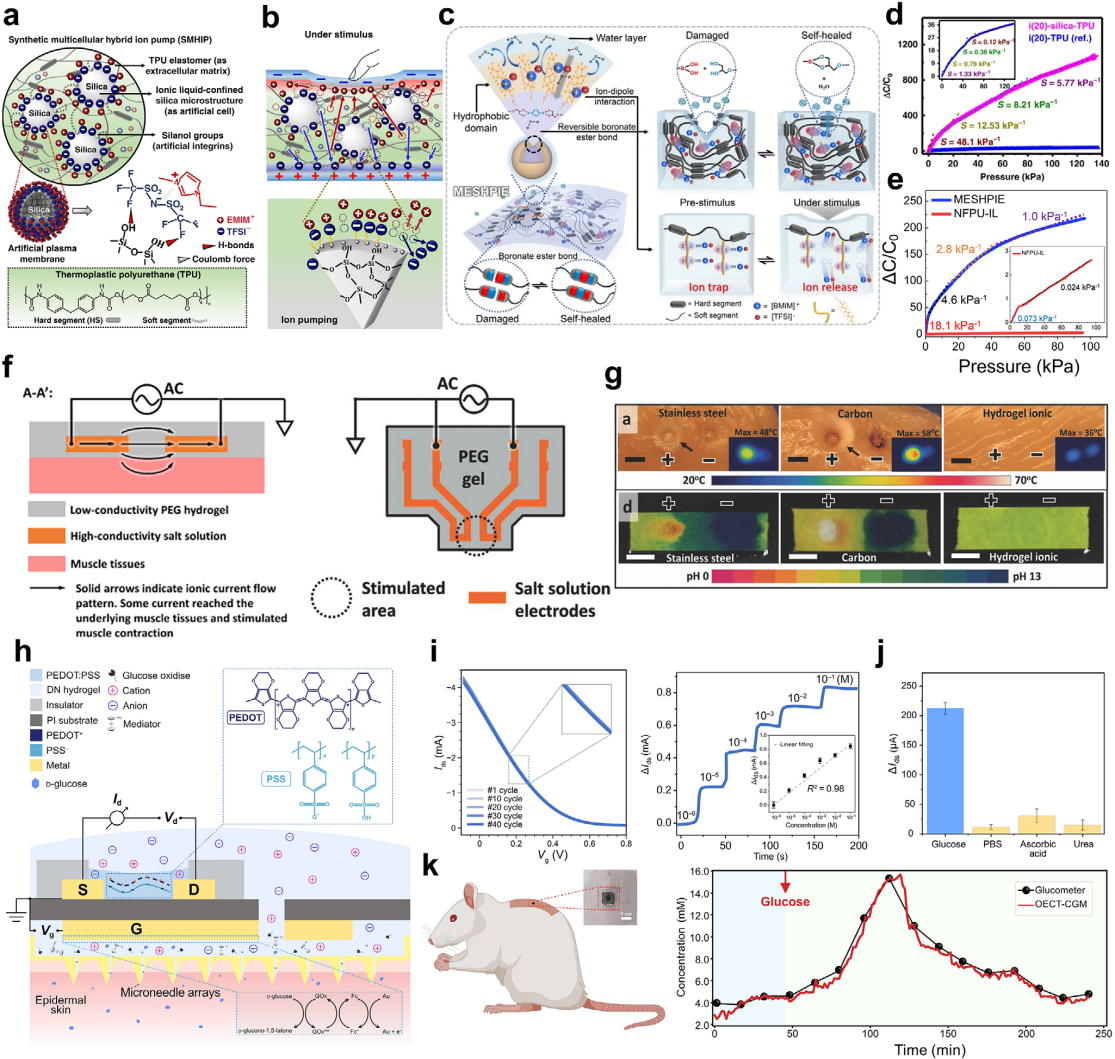
a) Design of a SMHIP consisting of [EMIM⁺][TFSI^−^]‐based ion pairs confined on the surface of silica microstructures dispersed in a TPU elastomer matrix. b) Mechanism of ion pumping from silica surfaces in SMHIP, triggered exclusively by pressure‐induced deformation. c) Schematic of MESHPIE with hydrophobic domains and reversible boronate bonds for self‐healing. d) Comparison of pressure sensitivity between i(20)–silica–TPU SMHIP and i(20)–TPU. e) Pressure sensitivity comparison of MESHPIE and NFPU‐IL under a 100 mV bias at 20 Hz. f) Schematic of a PEG hydrogel‐based soft electrode with a salt solution, illustrating ionic current flow for muscle stimulation. g) Comparison of thermal and pH effects of current injections using stainless‐steel, carbon, and hydrogel ionic electrodes. h) Schematic of the microneedle‐integrated OECT glucose sensor with a hydrogel electrolyte and a sensing mechanism. i) Transfer curves confirming the operational stability of the OECT device. j) Selective response of the OECT glucose sensor. k) In vivo evaluation of OECT‐CGM in a rat model, showing real‐time glucose monitoring comparable to a commercial glucometer. (a‐b) Reproduced with permission.^[^ ^151^ ^]^ Copyright 2019, Springer Nature. (c‐d) Reproduced with permission.^[^ ^152^ ^]^ Copyright 2024, Springer Nature. (f–g) Reproduced with permission.^[^ ^155^ ^]^ Copyright 2018, Wiley‐VCH. (h‐k) Reproduced with permission.^[^ ^158^ ^]^ Copyright 2024, American Association for the Advancement of Science.

To ensure both accurate sensing of subtle physiological cues and stable therapeutic outcomes, bioelectronic devices must provide precise electrical control within biological environments. In this context, achieving high sensitivity to specific stimuli is essential. Ion‐conducting gels, which transduce ionic dynamics into electrical signals, offer an effective platform for such applications. By engineering the ion transport and relaxation behavior within these gels, high‐performance devices with tunable electrical responses can be realized. A representative example involves the integration of silica particles into a thermoplastic polyurethane (TPU) matrix, where [EMIM⁺][TFSI^−^] ion pairs are immobilized via hydrogen bonding (**Figure** **9**a).^[^ ^170^ ^]^ As shown in Figure 9b, this design enables reversible ion trapping within the matrix, allowing for controlled ion release upon external stimulation. Such ion dynamics significantly enhance the sensitivity of the resulting sensor, enabling precise detection of mechanical stimuli. In a related approach, the incorporation of hydrophobic fluorinated chains enabled ion confinement through ion–dipole interactions, while the introduction of boronate groups imparted underwater self‐healing capabilities (Figure 9c).^[^ ^171^ ^]^ This molecular design strategy led to the development of ion‐conducting gels with high sensitivity and robust performance under diverse environmental conditions. Two pressure sensors engineered with controlled ion dynamics exhibited outstanding performance, achieving maximum sensitivities of 48.1 kPa^−1^ (Figure 9d) and 18.1 kPa^−1^ (Figure 9e), respectively. These values significantly surpass those of conventional ion gels, underscoring the effectiveness of ion confinement strategies in enhancing pressure responsiveness.

Beyond sensing, ion‐conducting gels are gaining attention as promising materials for biocompatible and stable bioelectric stimulators.^[^ ^95^, ^130^, ^148^ ^]^ Traditional metal electrodes are prone to inducing biological side effects such as pH fluctuations and thermal damage during long‐term use, highlighting the need for alternative stimulation interfaces.^[^ ^172^ ^]^ One such system uses a PEG‐based aqueous solution with high salt content to induce phase separation and form an ion gel electrode capable of delivering stimulation via internal ion transport without requiring a metal electrode (Figure 9f).^[^ ^173^ ^]^ Comparative studies of thermal and pH responses following current injection revealed that, unlike stainless steel and carbon electrodes, the ion gel electrode caused neither heat buildup nor pH shift at the stimulation site, confirming its thermal and chemical stability (Figure 9g).

In addition to high sensitivity and biostability, ion‐conducting gels enable low‐voltage operation, thus enhancing the longevity of implantable devices.^[^ ^174^, ^175^ ^]^ The microneedle‐integrated OECT sensor comprises a PEDOT:PSS‐based semiconductor channel and a multi‐network ion‐conductive gel electrolyte, enabling continuous glucose monitoring through a transdermal microneedle array (Figure 9h).^[^ ^176^ ^]^ This device operates stably at gate voltages below 0.6 V and maintains consistent current responses under repeated electrical cycling, confirming its robust operational reliability (Figure 9i). It also retains high selectivity for glucose, even in the presence of biological interferents such as PBS and urea (Figure 9j). In vivo testing further confirmed that the device achieved real‐time continuous glucose monitoring with an accuracy comparable to commercial glucometers (Figure 9k). Quantitative metrics for this device class are summarized in **Table** **2**, with the application domains and performance reported as a function of the ion‐conducting gel employed.

**Table 2:** Summary of device performance of sustainable bioelectronics.

Classification	Application	Performance	Ref
Hydrogel	Sensor	Power density: 0.85 µW cm^−3^	[1]
		SNR: 60(Glucose), LoD: 2 mm(Glucose)	[176]
		ECG monitoring without QRS vector inversion	[155]
		SNR: 60–100(ECG)	[189]
Iongel		Power consumption: <1 mV S: 48.1–5.77 kPa^−1^(0–135 kPa)	[170]
		S: 18.1–1.0 kPa^−1^(0–95 kPa)	[171]
		GF: 2.19, SNR: 8.91(ECG) and 5.63(EMG)	[141]
		S: 10.4 K^−1^	[181]
		LoD: 0.13 mV(K^+^), Stability: stable for 50 days	[187]
Eutectic gel		SNR: 36(EMG)	[159]
Hydrogel	Nerve stimulator	Activation of rat limb muscle at 2.5 V with 1.38 N	[173]
Iongel		Activation of rat limb muscle at 2.5 V with 1.38 N	[202]
Electrode: hydrogel OECT: iongel		Power consumption: 23.8 µW(on) and 7.96 µW(off) Activation of rat limb muscle at 1.0 V with 412 mN	[198]
Hydrogel	Supercapacitor	Power density: 1200 µW cm^−2^,	[144]
Iongel	Wound healing	Healing of rat calvarial defect within 2 weeks	[151]

GF: gauge factor;

SNR: signal to noise ratio;

LoD: limit of detection;

S: sensitivity.

Collectively, these findings demonstrate that ion‐conducting gels offer a next‐generation platform for both sensing and stimulation by integrating high sensitivity, biocompatibility, and low‐voltage operability, underscoring their potential as a key material for future wearable and implantable bioelectronic systems.

### 3.4 Environmental Robustness

In biological systems, a multitude of signals, including periodic and aperiodic inputs, are continuously present,^[^ ^177^ ^]^ among which some serve as meaningful targets, while others function as background noise.^[^ ^178^ ^]^ In addition, fluctuations in temperature, pressure, and ion concentration may further interfere with reliable device operation.^[^ ^179^ ^]^ This section highlights recent strategies designed to distinguish target signals from complex biological environments by minimizing signal and ionic interference. These include methods for decoupling specific mechanical or chemical stimuli from multisignal inputs, discriminating target ionic species within mixed electrolyte conditions, and improving signal‐to‐noise ratios under dynamic physiological conditions.^[^ ^180^ ^]^

**Figure 10:**
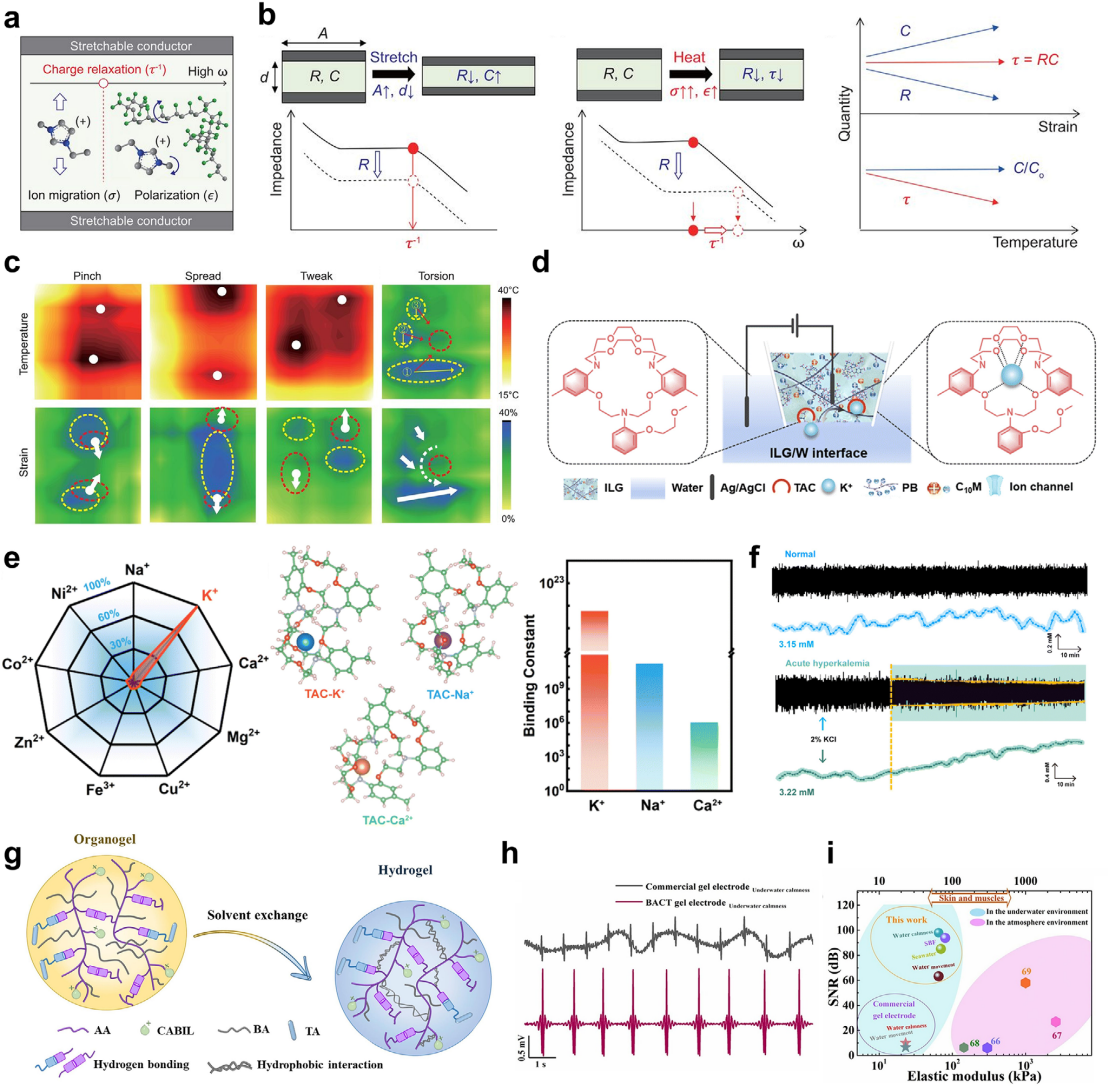
a) Frequency‐dependent behaviors of free ions in the alternating electric field. b) Bode plot scheme showing a parallel downshift of resistance (R) without a change in the charge relaxation frequency (τ−1) under stretching, and a decrease in R with an increase in τ−1 under heating. c) Pictures of the IEM‐skin under stimulations involving multiple stresses (pinch, spread, tweak, torsion) and temperature/strain profiles. In the strain profiles, the contact region (white dots), strained region (yellow dashed circles), heated region (red dashed circles), and the force vector (solid white arrows) are shown. d) Working principle of the molecularly tailored ILG‐TAC/W interface supported by a micropipette for the K^+^ ion‐selective transfer process. e) Ion selectivity tests of the ILG‐TAC electrode for metal cations (10 mM for Na^+^, 1 mM for Ca^2+^ and Mg^2+^, and 10 µM for other ions) and DFT simulations of binding between TAC and K^+^, Ni^2+^ and Ca^2+^. The calculated binding constant shows the highest interaction between TAC and K^+^ ions. f) Normal and acute hyperkalemia in the brains of mice, clearly identified by the ILG‐TAC microelectrode. g) The preparation process of the BACT gel electrode via a solvent exchange strategy for underwater sensing. h) ECG testing of the BACT gel electrode and commercial gel electrode in the calm underwater environment. BACT gel electrode shows a clear PQRST peak with a stable baseline. i) Comparison of the SNR and elastic modulus between the BACT gel electrode and other electrodes (in the underwater and atmospheric environment). (a‐c) Reproduced with permission.^[^ ^162^ ^]^ Copyright 2020, American Association for the Advancement of Science. (d‐f) Reproduced with permission.^[^ ^168^ ^]^ Copyright 2025, Royal Society of Chemistry. (g‐i) Reproduced with permission.^[^ ^170^ ^]^ Copyright 2022, Elsevier.

A multimodal sensory receptor was recently developed to precisely detect temperature and pressure stimuli by monitoring changes in ion relaxation time (τ^−1^ = ɛ/σ). As illustrated in **Figure** **10**a, ions in ion‐conducting gels exhibit both migration and polarization behaviors, depending on the frequency of the applied AC voltage. At lower frequencies, extended time allows a transition from polarization‐dominated to migration‐dominated behavior.^[^ ^181^ ^]^ The crossover point, defined as the ion relaxation time, exhibits distinct frequency shifts in response to temperature and pressure changes, thereby enabling effective decoupling of the two stimuli. As illustrated in Figure 10b, applying pressure reduces the electrode spacing, lowering both resistance and capacitance and thereby shifting the Bode plot downward. However, τ remains stable due to compensatory changes in ε and σ.^[^ ^182^ ^]^ In contrast, elevated temperature increases ionic mobility, resulting in reduced resistance and a similar downward shift in impedance. Additionally, migration behavior emerges at higher frequencies, shifting τ toward higher frequency regions. Capacitance served as a mechanical‐sensitive parameter that remained unaffected by temperature when calibrated against a reference value. In parallel, ion relaxation time (τ) was utilized as a temperature‐sensitive variable independent of mechanical strain, enabling effective decoupling. As shown in Figure 10c, a multimodal sensor composed of [EMIM^+^][TFSI^−^] based ion conductor and SEBS elastomeric electrodes demonstrated successful decoupling of temperature and pressure under varying shear forces.

Beyond decoupling dynamic stimuli, reliable sensing in biological environments requires high selectivity^[^ ^183^, ^184^, ^185^ ^]^ toward specific target molecules within complex mixtures of endogenous biomarkers. To ensure accurate signal interpretation, strategies that isolate and detect specific analytes from competing biochemical species are essential.^[^ ^186^ ^]^ To enable selective detection of K⁺ among various competing species, a micropipette‐based interfacial sensor (ILG‐TAC/W) was developed, incorporating [2.2.3]‐triazacryptand (TAC) within an ionic liquid gel matrix composed of a hydrophobic ionic liquid (C_10_M) and poly(1‐butyl‐3‐vinylmethylsulfonyl)imide (PB) (Figure 10d).^[^ ^187^ ^]^ TAC exhibited strong binding affinity and high selectivity toward K⁺ over other metal ions and biomolecules (Figure 10e), enabling interference‐free sensing. The sensor accurately monitored potassium levels in mouse models of acute and chronic hyperkalemia, confirming its in vivo selectivity and sensing capability (Figure 10f). In addition to achieving selective detection under complex biological conditions, it is also essential to ensure stable and consistent performance under dynamic environments.^[^ ^183^ ^]^ That is, environmental robustness is defined not only by sensing capability, but by maintaining reliable device operation across diverse physiological conditions. Recent efforts have introduced ion‐conducting gel‐based devices that maintain a high signal‐to‐noise ratio and stable operation even in aqueous environments, which can closely reflect physiological conditions. As shown in Figure 10g, an ion‐conducting gel was fabricated using a dual‐network structure composed of acrylic acid (AA) and butyl acrylate (BA), incorporating choline acrylate‐based bio‐ionic liquid (CABIL) as the ionic conductor and forming adhesive interactions via tannic acid (TA).^[^ ^188^, ^189^, ^190^, ^191^ ^]^ DMSO was initially used as a solvent, followed by solvent exchange, which promoted aggregation of hydrophobic segments, resulting in enhanced underwater adhesion.

In Figure 10h, while commercial gel electrodes failed to maintain a stable baseline in water, the butyl acrylate‐Acrylic acid‐choline acrylate bio ionic liquid‐tannic acid (BA‐AA‐CABIL‐TA; BACT) conductive hydrogel achieved superior biosignal detection due to strong underwater adhesion.^[^ ^192^ ^]^ Moreover, as demonstrated in Figure 10i, the device exhibited consistent performance under various solvent conditions, highlighting its robustness for operation in dynamic environments. Collectively, these strategies underscore the importance of designing ion‐conducting gel systems that not only offer high selectivity and multimodal sensing capabilities but also sustain reliable performance under complex and dynamically changing physiological environments. For each gel type, the application and number of days of stable operation under the stated conditions are listed in **Table** **3**, together with the explicit stability criteria used in each study.

**Table 3:** Summary of environmental stability for sustainable bioelectronics.

Classification	Application	Performance	Ref
Hydrogel	Sensor	Stable for 30 days	[155]
Iongel		Stable for 50 days	[187]
Hydrogel		Stable for 15 days	[189]
Electrode : hydrogel Semiconductor: iongel	Nerve stimulator	Stable for 15 days	[198]
Hydrogel	Supercapacitor	Stable for 120 days and 20 000 cycle	[144]
Iongel	Wound healing	Stable for 19 days	[151]

This section consolidated device‐level strategies that sustain reliable operation at the tissue–electronics interface: i) biocompatibility, achieved by non‐cytotoxic formulations and minimized ion release to ensure interface safety; ii) interface stability, encompassing anti‐fouling behavior, robust adhesion without delamination, corrosion/oxidation resistance, and impedance stability in physiological media; iii) high‐performance electrical properties, including low‐voltage operation within the electrochemical window, resilient ion–electron coupling that preserves capacitance/transconductance and biosignal fidelity, and charge‐balanced stimulation that avoids faradaic by‐products; and iv) environmental robustness, demonstrated by stable SNR and underwater/aqueous performance via strong wet adhesion and tolerance to solvent and ionic fluctuations. Together, these guidelines establish a clear chain from device architecture to interface chemistry and mechanics to functional readout, providing actionable criteria for integration into closed‐loop systems that maintain durable function in vivo.

## 4 System Sustainability of Ion‐Conducting Gel Platforms for Closed‐Loop Bioelectronics

Informed by the above device‐level principles, the following section addresses system‐level integration, including signal processing, closed‐loop control, and translation under biological variability, which are required to sustain performance during long‐term operation.

To fully realize the potential of ion‐conducting gels in clinical bioelectronic applications, it is not sufficient to focus solely on the stability and performance of individual materials or devices. Instead, there is a critical need to develop integrated systems that ensure sustainable, long‐term functionality under dynamic physiological conditions.^[^ ^130^, ^193^ ^]^ A key objective is the creation of closed‐loop bioelectronic platforms that not only monitor biosignals with high fidelity but also deliver timely and precise therapeutic feedback.^[^ ^194^, ^195^ ^]^ This requires seamless integration of stable ionic conductors, responsive electronic interfaces, and adaptive signal processing circuits capable of bidirectional communication with biological systems. Recent efforts have begun to explore such system‐level designs, incorporating mechanically compliant materials, self‐healing interfaces, and energy‐efficient architectures that collectively support autonomous, real‐time physiological modulation.^[^ ^196^, ^197^ ^]^ In such systems, stable gel–tissue coupling and low‐impedance transduction at the device level translate into reliable sensing, actuation, and runtime decision‐making. Recent demonstrations, including stretchable neuromorphic or efferent‐nerve concepts that use ion‐gel synaptic transistors and peripheral neuromodulation with evoked‐response feedback, show that gel‐enabled interfaces contribute directly to closed‐loop performance, with benefits such as improved energy efficiency, reduced motion artifacts, and patient‐specific therapy optimization.^[^ ^1^, ^2^, ^3^, ^4^, ^5^, ^6^ ^]^ We highlight emerging strategies for achieving system sustainability by coupling robust ion‐conducting devices with intelligent control schemes, thereby advancing toward closed‐loop platforms for next‐generation bioelectronic medicine. The two referenced studies exemplify this approach by demonstrating the pivotal role of ion‐conducting gels in facilitating bioelectronic interfaces capable of real‐time bidirectional communication and closed‐loop control.

Recently, a stretchable neuromorphic efferent nerve (SNEN) integrates ion‐conducting gels as the dielectric medium within synaptic transistors, enabling low‐power, mechanically compliant neuromorphic signal processing that emulates biological nerve functions.^[^ ^198^ ^]^ The ion gel allows stable ionic‐electronic coupling under large strain, contributing to durable synaptic transistor operation during continuous mechanical deformation.^[^ ^199^, ^200^, ^201^ ^]^ This enables naturalistic muscle stimulation with proprioceptive feedback in vivo, forming a closed‐loop system where biosignals are sensed and used to modulate motor output with high fidelity and low power consumption. The SNEN architecture leverages ion‐conducting gels not only for ionic conduction but also as critical components ensuring interface stability and mechanical compliance necessary for reliable long‐term neuromodulation.

In addition, a fully implantable peripheral nerve stimulation system for bladder regulation employing flexible electrodes coated with a gelatin methacryloyl (GelMA)‐based ion‐conducting gel infused with choline malate ionic liquid.^[^ ^202^ ^]^ This ion‐conducting gel coating significantly enhances electrode‐tissue interfacing by increasing electrical double‐layer capacitance and providing mechanical compliance that matches neural tissue, resulting in improved signal fidelity and reduced impedance.^[^ ^203^, ^204^ ^]^ The closed‐loop system uses evoked compound action potential (ECAP) feedback recorded via these ion gel‐coated electrodes to dynamically adjust stimulation parameters. This feedback mechanism enables precise, patient‐specific neuromodulation with superior therapeutic outcomes compared to conventional open‐loop systems. The study highlights the ion gel's essential role in both stable neural interfacing and enabling adaptive, feedback‐driven control architectures suitable for chronic implantation.

In addition, complementing these hardware‐centric advances, data‐driven methods can act as cross‐cutting enablers that operationalize our sustainability criteria across scales. At the materials level, data‐centric inverse design can learn structure–property relationships across polymers and ionic liquids from curated datasets, then nominate candidates prior to synthesis so that electrochemical stability, low‐leaching surrogates, and baseline biocompatibility screens are enforced from the outset.^[^ ^205^, ^206^ ^]^ In practice, learned representations narrow the search space, prioritize chemistries with robust stability and ion retention under physiological conditions, and route a small set of high‐value formulations to wet‐lab validation. At the device level, learning‐based analysis of impedance and related spectra can automatically identify and parameterize interface models, reduce user bias, and shorten model selection; the same analytics support drift‐aware monitoring by detecting early parameter shifts, attributing likely causes (aging, fouling, moisture uptake), and triggering targeted recalibration or maintenance—moving routine operation toward predictive upkeep.^[^ ^207^ ^]^ At the system level, learning‐based closed‐loop controllers can decode user‐specific physiological states in real time and adapt sensing and stimulation policies on the fly, with safeguards such as rate limits and confidence gating, and with personalization routines (on‐device fine‐tuning or transfer) to maintain safety and efficiency over long horizons.^[^ ^208^ ^]^ In this framing, AI complements rather than replaces the principles articulated above by providing the practical machinery to implement them more efficiently, more consistently, and at scale.

Combined with these analytics, gel‐enabled architectures provide a practical route to sustained, closed‐loop operation in vivo. Together, these studies underscore the importance of ion‐conducting gels as foundational materials that not only ensure stable electrical and mechanical interfaces with biological tissues but also facilitate the integration of sensors, stimulators, and processing units into cohesive closed‐loop bioelectronic platforms. By coupling the inherent ionic conductivity and mechanical compliance of ion gels with intelligent control circuits and adaptive feedback, next‐generation bioelectronic systems can achieve autonomous, precise, and durable physiological modulation tailored to dynamic biological environments. This integration of ion gels within closed‐loop architectures is vital for advancing sustainable, clinically effective bioelectronics.

## 5 Outlook

The advancement of bioelectronics critically depends on the development of fully integrated platforms that synergistically combine material innovation, device engineering, and system‐level intelligence. Ion‐conducting gels have emerged as a transformative class of materials that bridge the biological and electronic domains, offering unmatched ionic conductivity, mechanical compliance, and biointerface stability. These unique properties position ion‐conducting gels as the molecular foundation upon which next‐generation bioelectronic devices can be constructed.

However, achieving clinical translation demands moving beyond isolated improvements in materials or devices. The true potential of ion‐conducting gels is realized only when they are embedded within high‐performance devices capable of reliable biosignal transduction and safe electrical stimulation, which in turn are integrated into closed‐loop systems capable of real‐time physiological monitoring and autonomous feedback control. This hierarchical integration from molecular to systemic levels is essential to address the dynamic complexity and variability inherent in living systems.

Future efforts must therefore prioritize scalable manufacturing techniques for ion‐conducting gels with precisely tunable physicochemical properties, enabling reproducible device fabrication with consistent performance. Concurrently, device architectures must be refined to optimize ion‐electron coupling, minimize power consumption, and enhance mechanical robustness for chronic implantation. At the system level, incorporation of intelligent signal processing, machine learning algorithms, and adaptive control circuits will be critical to realize autonomous closed‐loop operation that can adjust therapeutic interventions in response to fluctuating biological signals.

Complementing these directions, data‐centric and physics‐informed AI can enhance sustainability across the material–device–system stack without shifting the focus away from core bioelectronics. At the material level, chemistry‐aware models with active learning accelerate the discovery of ion‐conducting gel chemistries that jointly expand the electrochemical window, reduce ion leaching, and preserve biocompatibility with fewer experiments and less waste.^[^ ^209^, ^210^ ^]^ At the device level, uncertainty‐aware digital twins predict drift and failure under physiological stress, guiding low‐voltage operation and architectures that prolong functional lifetime.^[^ ^211^ ^]^ At the system level, lightweight edge inference and reinforcement learning enable energy‐aware, closed‐loop control that adapts to biological variability while minimizing power and off‐target activation.^[^ ^212^ ^]^ Embedding these AI tools within standardized testing and life‐cycle assessment establishes a virtuous, multi‐objective optimization loop—balancing performance, safety, durability, and environmental impact.

In summary, the convergence of ion‐conducting gel materials with sophisticated device designs and intelligent system architectures heralds a paradigm shift in bioelectronics. By embracing a holistic platform‐based approach, future bioelectronic technologies will deliver precise, personalized, and sustainable healthcare solutions, ultimately transforming patient outcomes and quality of life.

## 6 Conclusion and Perspectives

Ion‐conducting gels have become essential materials bridging the biological and electronic realms, providing exceptional ionic conductivity, mechanical compliance, and biointerface compatibility. These attributes position them as critical components in the development of next‐generation bioelectronic devices designed for soft, conformable, and stable interfaces with living tissues. Despite considerable progress in material innovation, device engineering, and early‐stage system integration, challenges persist in achieving long‐term electrochemical stability, mechanical robustness, and reliable biointegration under the dynamic conditions of physiological environments. Overcoming these challenges necessitates an integrated approach that connects material design, device fabrication, and system‐level functionality.

Looking forward, the future of ion‐conducting gels in bioelectronics lies in scalable and reproducible material synthesis with precisely tunable ionic, mechanical, and biochemical properties suited for diverse biomedical applications. Device architectures must evolve to emphasize energy efficiency, miniaturization, and mechanical resilience to support chronic implantation and wearable technologies. At the system level, embedding adaptive control algorithms, real‐time analytics, and wireless communication will be essential to realize autonomous closed‐loop platforms capable of personalized therapy and continuous monitoring. Interdisciplinary collaborations across materials science, bioengineering, and clinical research will accelerate translation from laboratory innovation to practical healthcare solutions. With deeper insights into ion transport dynamics and biointerface interactions, novel strategies to mitigate biofouling, immune responses, and material degradation will emerge, paving the way for bioelectronics to fulfill its promise of delivering precise, reliable, and sustainable healthcare interventions.

## Conflict of Interest

The authors declare no conflict of interest.

## Author Contributions

D.H.K., T.K., and Ji Hong Kim proposed the story direction. Ji Hong Kim, W.H.C., and Jong Hwi Kim designed the outline and wrote the manuscript. W.H.C., Jong Hwi Kim, Y.P., and S.Y. provided the inspiration for the figures. D.H.K. supervised the whole research. The paper was edited by all the authors. All authors participated in the discussion and reviewed the manuscript before submission.

## References

[1] Y. Dobashi , D. Yao , Y. Petel , T. N. Nguyen , M. S. Sarwar , Y. Thabet , C. L. W. Ng , E. Scabeni Glitz , G. T. M. Nguyen , C. Plesse , F. Vidal , C. A. Michal , J. D. W. Madden , Science 2022, 376, 502. 35482868 10.1126/science.aaw1974

[2] J.‐S. Kim , J. Kim , J. Ahn , S. Chung , C.‐S. Han , Adv. Sci. 2023, 10, 2301037. 10.1002/advs.202301037 PMC10238195 37026619

[3] Y. H. Jung , B. Park , J. U. Kim , T.‐I. Kim , Adv. Mater. 2019, 31, 1803637. 10.1002/adma.201803637 30345558

[4] Y. Lin , A. Wu , Y. Zhang , H. Duan , P. Zhu , Y. Mao , Discover Nano 2025, 20, 60. 40156703 10.1186/s11671-025-04240-8 PMC11954787

[5] Y Huang , K. Yao , Q. Zhang , X. Huang , Z. Chen , Yu Zhou , X. Yu , Chem. Soc. Rev. 2024, 53, 8632. 39132912 10.1039/d4cs00413b

[6] V. Amoli , J. S. Kim , S. Y Kim , J. Koo , Y. S. Chung , H. Choi , D. H. Kim , Adv. Funct. Mater. 2020, 30, 1904532.

[7] D. T. Simon , E. O. Gabrielsson , K. Tybrandt , M. Berggren , Chem. Rev. 2016, 116, 13009. 27367172 10.1021/acs.chemrev.6b00146

[8] A. Zhang , C. M. Lieber , Chem. Rev. 2016, 116, 215. 26691648 10.1021/acs.chemrev.5b00608 PMC4867216

[9] P. Li , S. Kim , B. Tian , Device 2024, 2, 100401. 39119268 10.1016/j.device.2024.100401 PMC11308927

[10] B. A. Miao , L. Meng , B. Tian , Nanoscale Horiz. 2022, 7, 94. 34904138 10.1039/d1nh00538c

[11] W. Muhammad , J. Song , S. Kim , F. Ahmed , E. Cho , H. Lee , J. Kim , Biosensors 2025, 15, 119. 39997021 10.3390/bios15020119 PMC11852904

[12] K. D. Wise , IEEE Eng. Med. Biol. Mag. 2005, 24, 22. 10.1109/memb.2005.1511497 16248114

[13] M. Athanasopoulos , P. Samara , I. Athanasopoulos , Encyclopedia 2024, 4, 125.

[14] J. Gardner , Social Stud. Sci. 2013, 43, 707.

[15] A. Sarem‐Aslani , K. Mullett , Front. Integr. Neurosci. 2011, 5, 46. 21991248 10.3389/fnint.2011.00046 PMC3180671

[16] P. Sahu , S. Acharya , M. Totade , Cureus 2023, 15, 46389. 10.7759/cureus.46389 PMC10620620 37927638

[17] A. Yu , M. Zhu , C. Chen , Y. Li , H. Cui , S. Liu , Q. Zhao , Adv. Healthcare Mater. 2024, 13, 2302460. 10.1002/adhm.202302460 37816513

[18] Y. Liu , Z. Liu , Y. Tian , Acc. Chem. Res. 2022, 55, 2821. 36074539 10.1021/acs.accounts.2c00333

[19] G. S. Wilson , R. Gifford , Biosens. Bioelectron. 2005, 20, 2388. 15854814 10.1016/j.bios.2004.12.003

[20] F. Jin , T. Li , Z. Wei , R. Xiong , L. Qian , J. Ma , T. Yuan , Qi Wu , C. Lai , X. Ma , F. Wang , Y. Zhao , F. Sun , T. Wang , Z‐Qi Feng , Nat. Commun. 2022, 13, 5302. 36085331 10.1038/s41467-022-33089-z PMC9463164

[21] E. Kim , S. Kim , Y. W. Kwon , H. Seo , M. Kim , W. G. Chung , W. Park , H. Song , D. H. Lee , J. Lee , S. Lee , I. Jeong , K. Lim , J.‐U. Park , Interdiscipl. Med. 2023, 1, 20230003.

[22] J. M. A. Tanskanen , A. Ahtiainen , J. A. K. Hyttinen , Bioelectricity 2020, 2, 328. 34471853 10.1089/bioe.2020.0028 PMC8370352

[23] Y. H. Jung , J. U. Kim , J. S. Lee , J. H. Shin , W. Jung , J. Ok , T‐Il Kim , Adv. Mater. 2020, 32, 1907478. 10.1002/adma.201907478 32104960

[24] K. J. Yu , Z. Yan , M. Han , J. A. Rogers , npj Flexible Electron. 2017, 1, 4.

[25] S. H. Lee , C. K. Jeong , G.‐T. Hwang , K. J. Lee , Nano Energy 2015, 14, 111.

[26] H. J. Lee , Y. Son , J. Kim , C. J Lee , E.‐S. Yoon , Il‐J Cho , Lab Chip 2015, 15, 1590. 25651943 10.1039/c4lc01321b

[27] Y. Chen , Y. Zhang , Z. Liang , Yu Cao , Z. Han , X. Feng , npj Flexible Electron. 2020, 4, 2.

[28] S. Oh , S. Lee , S. W. Kim , C. Y. Kim , E. Y. Jeong , J. Lee , D. A. Kwon , J.‐W. Jeong , Biosens. Bioelectron. 2024, 258, 116328. 38692223 10.1016/j.bios.2024.116328

[29] E. Mazza , A. E. Ehret , J. Mech. Behav. Biomed. Mater. 2015, 48, 100. 25916818 10.1016/j.jmbbm.2015.03.023

[30] L. Wang , C. Zhang , Z. Hao , S. Yao , L. Bai , J. M. Oliveira , P. Wang , K. Zhang , C. Zhang , J. He , R. L. Reis , D. Li , Bioact. Mater. 2025, 47, 18. 39872211 10.1016/j.bioactmat.2024.12.033 PMC11762938

[31] Y. Qian , X. Zhang , L. Xie , D. Qi , B. K. Chandran , X. Chen , W. Huang , Adv. Mater. 2016, 28, 9243. 27573694 10.1002/adma.201601278

[32] N. D. Treat , P. Westacott , N. Stingelin , Annu. Rev. Mater. Res. 2015, 45, 459.

[33] H. Liu , D. Liu , J. Yang , H. Gao , Y. Wu , Small 2023, 19, 2206938. 10.1002/smll.202206938 36642796

[34] C. Wang , H. Dong , L. Jiang , W. Hu , Chem. Soc. Rev. 2018, 47, 422. 29186226 10.1039/c7cs00490g

[35] Y. Shin , H. S. Lee , Y. J. Hong , S.‐H. Sunwoo , O. K. Park , S. H. Choi , D.‐H. Kim , S. Lee , Sci. Adv. 2024, 10, adi7724. 10.1126/sciadv.adi7724 PMC10954228 38507496

[36] S. Inal , J. Rivnay , A.‐O. Suiu , G. G. Malliaras , I. McCulloch , Acc. Chem. Res. 2018, 51, 1368. 29874033 10.1021/acs.accounts.7b00624

[37] X. Gao , Y. Bao , Z. Chen , J. Lu , T. Su , L. Zhang , J. Ouyang , Adv. Electron. Mater. 2023, 9, 2300082.

[38] Z. Wang , H. Bai , W. Yu , Z. Gao , W. Chen , Z. Yang , C. Zhu , Y. Huang , F. Lv , S. Wang , Sci. Adv. 2022, 8, abo1458. 10.1126/sciadv.abo1458 PMC9216517 35731871

[39] Y. Jiang , Z. Zhang , Yi‐X Wang , D. Li , C.‐T. Coen , E. Hwaun , G. Chen , H.‐C. Wu , D. Zhong , S. Niu , W. Wang , A. Saberi , J.‐C. Lai , Y. Wu , Y. Wang , A. A. Trotsyuk , K. Y. Loh , C.‐C. Shih , W. Xu , K. Liang , K. Zhang , Y. Bai , G. Gurusankar , W. Hu , W. Jia , Z. Cheng , R. H. Dauskardt , G. C. Gurtner , J. B.‐H. Tok , K. Deisseroth , et al., Science 2022, 375, 1411. 35324282 10.1126/science.abj7564

[40] S. Nambiar , J. T. W. Yeow , Biosens. Bioelectron. 2011, 26, 1825. 21030240 10.1016/j.bios.2010.09.046

[41] M. Jia , M. Rolandi , Adv. Healthcare Mater. 2020, 9, 1901372.

[42] F. Fu , J. Wang , H. Zeng , J. Yu , ACS Mater. Lett. 2020, 2, 1287.

[43] H. Yuk , B. Lu , X. Zhao , Chem. Soc. Rev. 2019, 48, 1642. 30474663 10.1039/c8cs00595h

[44] Z. Li , J. Lu , T. Ji , Y. Xue , L. Zhao , K. Zhao , B. Jia , B. Wang , J. Wang , S. Zhang , Z. Jiang , Adv. Mater. 2024, 36, 2306350. 10.1002/adma.202306350 37987498

[45] X. Lyu , Y. Hu , S. Shi , S. Wang , H. Li , Y. Wang , K. Zhou , Biosensors 2023, 13, 815. 37622901 10.3390/bios13080815 PMC10452556

[46] D. Gao , S. Fabiano , Science 2024, 384, 509. 38696588 10.1126/science.adp3192

[47] Y. Li , S. Tan , X. Zhang , Z. Li , J. Cai , Y. Liu , Gels 2025, 11, 258. 40277694 10.3390/gels11040258 PMC12027214

[48] G. Tian , D. Yang , C. Liang , Y. Liu , J. Chen , Q. Zhao , S. Tang , J. Huang , P. Xu , Z. Liu , D. Qi , Adv. Mater. 2023, 35, 2212302. 10.1002/adma.202212302 36739173

[49] C. Xie , X. Wang , H. He , Y. Ding , X. Lu , Adv. Funct. Mater. 2020, 30, 1909954.

[50] H.‐R. Lee , C.‐C. Kim , J.‐Y. Sun , Adv. Mater. 2018, 30, 1704403. 10.1002/adma.201704403 29889329

[51] I. K. Han , K.‐I. Song , S.‐M. Jung , Y. Jo , J. Kwon , T. Chung , S. Yoo , J. Jang , Y.‐T. Kim , D. S. Hwang , Y. S. Kim , Adv. Mater. 2023, 35, 2203431. 10.1002/adma.202203431 35816086

[52] J. Zhang , L. Wang , Yu Xue , I. M. Lei , X. Chen , P. Zhang , C. Cai , X. Liang , Yi Lu , Ji Liu , Adv. Mater. 2023, 35, 2209324. 10.1002/adma.202209324 36398434

[53] J. G. Roth , M. S. Huang , T. L. Li , V. R. Feig , Y. Jiang , B. Cui , H. T. Greely , Z. Bao , S. P. Pasca , S. C. Heilshorn , Nat. Rev. Neurosci. 2021, 22, 593. 34376834 10.1038/s41583-021-00496-y PMC8612873

[54] S. Park , H. Yuk , R. Zhao , Y. S. Yim , E. W. Woldeghebriel , J. Kang , A. Canales , Y. Fink , G. B. Choi , X. Zhao , P. Anikeeva , Nat. Commun. 2021, 12, 3435. 34103511 10.1038/s41467-021-23802-9 PMC8187649

[55] A. N. Dalrymple , U. A. Robles , M. Huynh , B. A. Nayagam , R. A. Green , L. A. Poole‐Warren , J. B. Fallon , R. K. Shepherd , J. Neural Eng. 2020, 17, 026018. 32135529 10.1088/1741-2552/ab7cfc

[56] Y. Ouyang , X. Li , S. Li , Z. L. Wang , Di Wei , ACS Appl. Mater. Interfaces 2024, 16, 18236. 38536118 10.1021/acsami.4c02303

[57] K. Jia , X. Li , Y. Wang , Soft Matter 2021, 17, 834. 33325974 10.1039/d0sm01789b

[58] H. Yan , R. Qi , Z. Liu , H. Wang , C. Dong , Li‐Z Zhang , Device 2025, 3, 100568.

[59] C. Yang , Z. Suo , Nat. Rev. Mater. 2018, 3, 125.

[60] K. Kadan‐Jamal , F. Wronowski , A. Jin , T. E. Naegele , V. R. Montes , X. Tao , A. Dominguez‐Alfaro , C. Lee , G. G. Malliaras , Chem. Rev. 2025, 125, 6874. 40674565 10.1021/acs.chemrev.4c00468 PMC12355715

[61] J. Poe , S. Sriram , Y. Mehkri , B. Lucke‐Wold , CNS Neurol. Disord.‐Drug Targets 2024, 23, 841. 36790006 10.2174/1871527322666230215144649

[62] E. Alidoosti , H. Zhao , Langmuir 2018, 34, 5592. 29688021 10.1021/acs.langmuir.8b00855 PMC5953854

[63] M. C. Koetting , J. T. Peters , S. D. Steichen , N. A. Peppas , Mater. Sci. Eng. R Rep. 2015, 93, 1. 27134415 10.1016/j.mser.2015.04.001 PMC4847551

[64] T. Benselfelt , J. Shakya , P. Rothemund , S. B. Lindström , A. Piper , T. E. Winkler , A. Hajian , L. Wågberg , C. Keplinger , M. M. Hamedi , Adv. Mater. 2023, 35, 2303255. 10.1002/adma.202303255 37451686

[65] M. M. H. Rumon , RSC Adv. 2025, 15, 11688. 40236573 10.1039/d5ra00521c PMC11997669

[66] S. Khattak , I. Ullah , M. Sohail , M. U. Akbar , M. A. Rauf , S. Ullah , J. Shen , H.‐T. Xu , Aggregate 2025, 6, 688.

[67] E. M. Ahmed , J. Adv. Res. 2015, 6, 105.25750745

[68] S. Correa , A. K. Grosskopf , H. Lopez Hernandez , D. Chan , A. C. Yu , L. M. Stapleton , E. A. Appel , Chem. Rev. 2021, 121, 11385. 33938724 10.1021/acs.chemrev.0c01177 PMC8461619

[69] J. Ehlich , C. Vašíček , J. Dobes , A. Ruggiero , M. Vejvodová , E. D. Glowacki , ACS Appl. Mater. Interfaces 2024, 16, 53567. 39351783 10.1021/acsami.4c12268 PMC11472339

[70] P. Lu , D. Ruan , M. Huang , Mi Tian , K. Zhu , Z. Gan , Z. Xiao , Signal Transduct. Target. Ther. 2024, 9, 166. 38945949 10.1038/s41392-024-01852-x PMC11214942

[71] X. S. Zheng , C. Tan , E. Castagnola , X. T. Cui , Adv. Healthcare Mater. 2021, 10, 2100119. 10.1002/adhm.202100119 PMC8257249 34029008

[72] R. Vatsyayan , D. Cleary , J. R. Martin , E. Halgren , S. A. Dayeh , J. Neural Eng. 2021, 18, 046077. 10.1088/1741-2552/ac038b PMC8216108 34015769

[73] M. Görlin , P. Chernev , J. Ferreira de Araújo , T. Reier , S. Dresp , B. Paul , R. Krähnert , H. Dau , P. Strasser , J. Am. Chem. Soc. 2016, 138, 5603. 27031737 10.1021/jacs.6b00332

[74] S. F. Cogan , K. A. Ludwig , C. G. Welle , P. Takmakov , J. Neural Eng. 2016, 13, 021001. 26792176 10.1088/1741-2560/13/2/021001 PMC5386002

[75] E. M. Hudak , D. W. Kumsa , H. B. Martin , J. T. Mortimer , J. Neural Eng. 2017, 14, 046012. 28345534 10.1088/1741-2552/aa6945 PMC5728108

[76] G. Salvi , P. De Los Rios , M. Vendruscolo , Proteins: Struct., Funct., Bioinf. 2005, 61, 492. 10.1002/prot.20626 16152629

[77] M. McLaughlin , M. J. Earle , M. A. Gîlea , B. F. Gilmore , S. P. Gorman , K. R. Seddon , Green Chem. 2011, 13, 2794.

[78] S. Ganguly , S. Margel , Gels 2025, 11, 219. 40277655 10.3390/gels11040219 PMC12026471

[79] T. Lv , L. Suo , Curr. Opin. Electrochem. 2021, 29, 100818.

[80] Y. Zhao , X. Hu , G. D. Stucky , S. W. Boettcher , J. Am. Chem. Soc. 2024, 146, 3438. 38288948 10.1021/jacs.3c12980

[81] A. M. O'Mahony , D. S. Silvester , L. Aldous , C. Hardacre , R. G. Compton , J. Chem. Eng. Data 2008, 53, 2884.

[82] M. Chen , J. Wu , T. Ye , J. Ye , C. Zhao , S. Bi , J. Yan , B. Mao , G. Feng , Nat. Commun. 2020, 11, 5809. 33199709 10.1038/s41467-020-19469-3 PMC7670447

[83] Y. Gu , X. Zheng , Z. Zhou , G. Chen , S. Chen , Q. Li , J. Energy Storage 2024, 89, 111892.

[84] Z. Qi , R. Ren , J. Hu , Y. Chen , Y. Guo , Y. Huang , J. Wei , H. Zhang , Q. Pang , X. Zhang , H. Wang , Small 2024, 20, 2400369. 10.1002/smll.202400369 38558327

[85] J. Chen , Y. Liu , F. Chen , M. Guo , J. Zhou , P. Fu , X. Zhang , X. Wang , He Wang , W. Hua , J. Chen , J. Hu , Y. Mao , D. Jin , W. Bu , Nat. Commun. 2024, 15, 405. 38195782 10.1038/s41467-023-44635-8 PMC10776784

[86] H. Li , J. Wang , Y. Fang , Microsyst. Nanoeng. 2023, 9, 4. 36620392 10.1038/s41378-022-00444-5 PMC9810608

[87] X. Ju , J. Kong , G. Qi , S. Hou , X. Diao , S. Dong , Y. Jin , Nat. Commun. 2024, 15, 762. 38278810 10.1038/s41467-024-45070-z PMC10817919

[88] D. Datta , V. Colaco , S. P. Bandi , N. Dhas , L. S. L. Janardhanam , S. Singh , L. K. Vora , ACS Biomater. Sci. Eng. 2025, 11, 1338. 39999055 10.1021/acsbiomaterials.4c02264 PMC11897956

[89] Z. Yan , W. Gao , Y. Duan , H. Zhou , C. Ni , Li Huang , Z. Ye , Bioact. Mater. 2025, 52, 634. 40607118 10.1016/j.bioactmat.2025.06.014 PMC12221386

[90] S‐Ha Jeong , Y. Lee , M.‐G. Lee , W. J. Song , Ji‐U Park , J.‐Y. Sun , Nano Energy 2021, 79, 105463.

[91] J.‐C. Heo , B. Kim , Y.‐N. Kim , D.‐K. Kim , J‐Ha Lee , Sensors 2017, 17, 2905.29240666

[92] Y Xin , B. Sun , Y. Kong , B. Zhao , J. Chen , K. Shen , Y. Zhang , Nanoscale 2025, 17, 2423. 39844771 10.1039/d4nr04645e

[93] D. Ho , ChemElectroChem 2024, 11, 202300268.

[94] J. W. Suen , N. K. Elumalai , S. Debnath , N. M. Mubarak , C. I. Lim , M. M. Reddy , Adv. Mater. Interfaces 2022, 9, 2201405.

[95] C. Jiao , J. Liu , S. Yan , Z. Xu , Z. Hou , W. Xu , J. Mater. Chem. C 2025, 13, 2620.

[96] G. Kaklamani , D. Kazaryan , J. Bowen , F. Iacovella , S. H. Anastasiadis , G. Deligeorgis , Regen. Biomater. 2018, 5, 293. 30338127 10.1093/rb/rby019 PMC6184632

[97] R. Sai , S. Hirata , H. Tsutsumi , Yu Katayama , Frontiers in Chemistry 2022, 10, 943224. 35910721 10.3389/fchem.2022.943224 PMC9329624

[98] Z. Shen , J. Ma , Y. Cai , S. Li , D. Ruan , S. Dai , Z. Sheng , J. Bai , D. Yin , J. Ping , Y. Ying , C. Yang , S. Qu , Z. Jia , Cell Rep. Phys. Sci. 2023, 4, 101741.

[99] Z. Zhao , H. Tu , E. G. R. Kim , B. F. Sloane , Y. Xu , Sens. Actuators, B 2017, 247, 92. 10.1016/j.snb.2017.02.135 PMC5621747 28970651

[100] X. He , B. Zhang , Q. Liu , H. Chen , J. Cheng , B. Jian , H. Yin , H. Li , Ke Duan , J. Zhang , Qi Ge , Nat. Commun. 2024, 15, 6431. 39085229 10.1038/s41467-024-50797-w PMC11291765

[101] M. Seitanidou , R. Blomgran , G. Pushpamithran , M. Berggren , D. T. Simon , Adv. Healthcare Mater. 2019, 8, 1900813. 10.1002/adhm.201900813 31502760

[102] S. Sen , S. Malunavar , A. J. Bhattacharyya , J. Phys. Chem. B 2016, 120, 10153. 27598796 10.1021/acs.jpcb.6b07523

[103] Y. He , Yu Cheng , C. Yang , C. F. Guo , Nat. Mater. 2024, 23, 1107. 38514845 10.1038/s41563-024-01848-6

[104] N. Li , Y. Li , Z. Cheng , Y. Liu , Y. Dai , S. Kang , S. Li , N. Shan , S. Wai , A. Ziaja , Y. Wang , J. Strzalka , W. Liu , C. Zhang , X. Gu , J. A. Hubbell , B. Tian , S. Wang , Science 2023, 381, 686. 37561870 10.1126/science.adg8758 PMC10768720

[105] S. J. K. O'Neill , Z. Huang , X. Chen , R. L. Sala , J. A. McCune , G. G. Malliaras , O. A. Scherman , Sci. Adv. 2024, 10, adn5142. 10.1126/sciadv.adn5142 PMC466958 39018406

[106] K. Tao , Z. Chen , J. Yu , H. Zeng , J. Wu , Z. Wu , Q. Jia , P. Li , Y. Fu , H. Chang , W. Yuan , Adv. Sci. 2022, 9, 2104168. 10.1002/advs.202104168 PMC8981453 35098703

[107] M. Xiao , J. Meng , H. Zhang , Z. Wu , Z. Li , Adv. Mater. 2025, 37, 06436. 10.1002/adma.202506436 40685875

[108] Z. Du , La Li , G. Shen , Nano‐Micro Lett. 2025, 17, 204. 10.1007/s40820-025-01721-4 PMC11958907 40163278

[109] N. Liu , H. Ma , M. Li , R. Qin , P. Li , FlexMat 2024, 1, 269.

[110] R. Liu , T. Wang , G. Li , Z. Fan , Q. Zhou , K. Wang , P. Li , W. Huang , Adv. Funct. Mater. 2023, 33, 2214917.

[111] W. Zhao , Y. Zheng , M. Jiang , T. Sun , A. Huang , L. Wang , W. Jiang , Q. Zhang , Sci. Adv. 2023, 9, adk2098. 10.1126/sciadv.adk2098 PMC10599631 37878706

[112] S. Y. Zheng , S. Mao , J. Yuan , S. Wang , X. He , X. Zhang , C. Du , D. Zhang , Z. L. Wu , J. Yang , Chem. Mater. 2021, 33, 8418.

[113] D. Ji , J. M. Park , M. S. Oh , T. L. Nguyen , H. Shin , J. S. Kim , D. Kim , H. S. Park , J. Kim , Nat. Commun. 2022, 13, 3019. 35641519 10.1038/s41467-022-30691-z PMC9156673

[114] R. Ji , S. Yan , Z. Zhu , Y. Wang , D. He , K. Wang , D. Zhou , Q. Jia , X. Wang , B. Zhang , C. Shi , T. Xu , R. Wang , R. Wang , Y. Zhou , Adv. Sci. 2024, 11, 2401869. 10.1002/advs.202401869 PMC11434023 38959395

[115] T. Shen , J. Kan , E. Benet , F. J. Vernerey , Soft Matter 2019, 15, 5842. 31290890 10.1039/c9sm00911f

[116] P. Heidarian , A. Z. Kouzani , Carbohydr. Polym. 2023, 313, 120879. 37182969 10.1016/j.carbpol.2023.120879

[117] Y. Liu , S. Shen , Z. Duan , J. Deng , D. Fan , Adv. Funct. Mater. 2025, 35, 2504356.

[118] Y. Jian , S. Handschuh‐Wang , J. Zhang , W. Lu , X. Zhou , T. Chen , Mater. Horiz. 2021, 8, 351. 34821259 10.1039/d0mh01029d

[119] S. A. Merchant , D. T. Glatzhofer , D. W. Schmidtke , Langmuir 2007, 23, 11295. 17902716 10.1021/la701521s

[120] P. Sánchez‐Cid , M. Jiménez‐Rosado , J. F. Rubio‐Valle , A. Romero , F. J. Ostos , M. Rafii‐El‐Idrissi Benhnia , V. Perez‐Puyana , Polymers 2022, 14, 272. 35054678 10.3390/polym14020272 PMC8781623

[121] Y. Huang , C. Hao , J. Li , Z. Bao , T. Liu , X. Wang , ACS Appl. Polym. Mater 2025, 7, 959.

[122] X. Guo , S. Zhang , S. Patel , X. Sun , Y.‐L. Zhu , Z. Wei , R. Wang , X. He , Z. Wang , C. Yu , S. C. Tan , Sci. Adv. 2025, 11, adv8523. 10.1126/sciadv.adv8523 PMC12083530 40378220

[123] H. Liu , S. Guan , P. Wang , X. Dong , Small 2025, 21, 2407870. 10.1002/smll.202407870 39905917

[124] W. Niu , Q. Tian , Z. Liu , X. Liu , Adv. Mater. 2023, 35, 2304157. 10.1002/adma.202304157 37345560

[125] C. Wang , Y. Liu , X. Qu , B. Shi , Q. Zheng , X. Lin , S. Chao , C. Wang , J. Zhou , Yu Sun , G. Mao , Z. Li , Adv. Mater. 2022, 34, 2105416. 10.1002/adma.202105416 35103354

[126] Y. Niu , H. Liu , R. He , M. Luo , M. Shu , F. Xu , Small 2021, 17, 2101151. 10.1002/smll.202101151 34013638

[127] X. Di , J. Hou , M. Yang , G. Wu , P. Sun , Mater. Horiz. 2022, 9, 3057. 36239123 10.1039/d2mh00456a

[128] J. S. Kim , J. Kim , J. W. Lim , D. J. Kim , J. I. Lee , H. Choi , H. Kweon , J. Lee , H. Yee , J. H. Kim , B. Kim , M. S. Kang , J. H. Jeong , S.‐M. Park , D. H. Kim , ACS Nano 2023, 17, 14706. 37498185 10.1021/acsnano.3c02637

[129] K. H Li , J. Deng , M. Guo , M. Cai , Y. Zhang , C. F. Guo , Adv. Mater. 2022, 34, 2200261. 10.1002/adma.202200261 35170097

[130] L. W Yang , W. Liu , W. Li , Y. Huang , O. Jin , L. Zhang , Y. Jiang , Z. Luo , Nat. Commun. 2024, 15, 7993. 39266583 10.1038/s41467-024-52418-y PMC11393409

[131] H. W Rivnay , K. Deisseroth , G. G. Malliaras , Sci. Adv. 2017, 3, 1601649. 10.1126/sciadv.1601649 PMC5466371 28630894

[132] A. L. H Stewart , M. Zelzer , M. Marlow , A. M. Piccinini , Cell Biochem. Funct. 2024, 42, 4097. 10.1002/cbf.4097 39010326

[133] Q. Fang , J. Yan , W. Wang , X. Wang , J. Chen , Y. Xu , X. Deng , P. Li , S. Fa , Q. Zhang , Yi Yan , Biomacromolecules 2025, 26, 1498. 39954297 10.1021/acs.biomac.4c01226

[134] J. Zhang , X. Mao , Q. Jia , R. Nie , Y. Gao , K. Tao , H. Chang , P. Li , W. Huang , npj Flexible Electron. 2024, 8, 60.

[135] J. Liang , R. Lv , M. Li , J. Chai , S. Wang , W. Yan , Z. Zheng , P. Li , Macromol. Biosci. 2024, 24, 2300302. 10.1002/mabi.202300302 37815522

[136] Y. Xing , Y. Hu , X. Zhang , D. Zheng , G. Ma , Y. Diao , H. Yue , W. Wei , S. Zhang , Nat. Commun. 2025, 16, 6929. 40721585 10.1038/s41467-025-62206-x PMC12304108

[137] G. Mugnaini , R. Gelli , L. Mori , M. Bonini , ACS Appl. Polym. Mater 2023, 5, 9192.

[138] T. T. T. Vo , T‐Yu Peng , T. H. Nguyen , T. N. H. Bui , C.‐S. Wang , W‐Ju Lee , Y.‐L. Chen , Y.‐C. Wu , I‐T Lee , Cell Commun. Signal. 2024, 22, 353. 38970072 10.1186/s12964-024-01726-3 PMC11225285

[139] O. A. Legonkova , N. O. Sultanova , V. V. Stafford , A. A. Zavitaeva , D. S. Kopitsyn , E. R. Tolboeva , A. M. Mahmydov , V. A. Vinokurov , G. A. Davydova , N. B. Svishcheva , K. Barbaro , J. V. Rau , Molecules 2024, 29, 3247. 39064826 10.3390/molecules29143247 PMC11279792

[140] Q. Lv , D. Zhou , Y. He , T. Xu , X. Qiu , J. Zeng , Bioactive Materials 2025, 49, 172. 40124599 10.1016/j.bioactmat.2025.01.013 PMC11929901

[141] T. K Shen , J. Yu , F. Nan , Z. Wu , B. Li , J. Li , W. W. Yu , Acta Biomater. 2025, 199, 154. 40311991 10.1016/j.actbio.2025.04.053

[142] L. Liao , J. Zhang , J. Ding , C. Xu , L. Zhu , Y. Hou , S. Li , J. Zhang , B. Wei , H. Wang , Molecules 2024, 29, 5728. 39683887 10.3390/molecules29235728 PMC11643890

[143] H. Montazerian , E. Davoodi , C. Wang , F. Lorestani , J. Li , R. Haghniaz , R. R. Sampath , N. Mohaghegh , S. Khosravi , F. Zehtabi , Y. Zhao , N. Hosseinzadeh , T. Liu , T. K. Hsiai , A. H. Najafabadi , R. Langer , D. G. Anderson , P. S. Weiss , A. Khademhosseini , W. Gao , Nat. Commun. 2025, 16, 3755. 40263282 10.1038/s41467-025-59045-1 PMC12015517

[144] E. Wang , M. Wu , L. Luo , Xi Cui , L. Xu , R. Luo , Y. Zou , T. Le , Y. Shan , Y. Quan , Y. Bai , Li Wu , Y. Hu , S. Cheng , J. Yang , C. Zhu , D. Yu , J. Ji , Y. Ren , D. Jiang , B. Shi , H. Feng , W. Hua , Z. Li , H. Ouyang , Device 2025, 3, 100724.

[145] W. Liu , R. Xie , J. Zhu , J. Wu , J. Hui , X. Zheng , F. Huo , D. Fan , npj Flexible Electron. 2022, 6, 68.

[146] A. Tessier , S. Zhuo , S. K. Ameri , Biosensors 2024, 14, 405.39194634

[147] F. Han , S. Chen , F. Wang , M. Liu , J. Li , H. Liu , Y. Yang , H. Zhang , D. Liu , R. He , W. Cao , X. Qin , F. Xu , Adv. Sci. 2025, 12, 2412726. 10.1002/advs.202412726 PMC12021042 39874215

[148] J. Chong , C. Sung , K. S. Nam , T. Kang , H. Kim , H. Lee , H. Park , S. Park , J. Kang , Nat. Commun. 2023, 14, 2206. 37072411 10.1038/s41467-023-37948-1 PMC10113367

[149] X. Li , Y. Sun , S. Wang , G. Tian , T. Yang , L. Huang , Y. Ao , B. Lan , J. Zhang , T. Xu , Y. Liu , L. Jin , W. Yang , W. Deng , Chem. Eng. J. 2024, 498, 155195.

[150] S. Chen , J. Xie , J. Liu , X. Huang , C. Wang , J. Mater. Sci. 2021, 56, 2725.

[151] C. Jiang , J. Fu , H. Zhang , Y. Hua , L. Cao , J. Ren , M. Zhou , F. Jiang , X. Jiang , S. Ling , Adv. Mater. 2025, 37, 2413028. 10.1002/adma.202413028 39632650

[152] S. Kondaveeti , G. Choi , S. C. Veerla , S. Kim , J. Kim , H. J. Lee , U. Kuzhiumparambil , P. J. Ralph , J. Yeo , H. E. Jeong , Nano Convergence 2024, 11, 12. 38512587 10.1186/s40580-024-00419-4 PMC10957857

[153] H. Tang , Y. Li , S. Liao , H. Liu , Y. Qiao , J. Zhou , Adv. Healthcare Mater. 2024, 13, 2400562. 10.1002/adhm.202400562 38773929

[154] G. J. Rodriguez‐Rivera , A. Post , M. John , S. Buchan , D. Bernard , M. Razavi , E. Cosgriff‐Hernandez , Nat. Commun. 2024, 15, 64. 38167848 10.1038/s41467-023-44419-0 PMC10762156

[155] G. J. Rodriguez‐Rivera , A. Post , M. John , D. Bashe , F. Xu , T. Larue , A. Nkansah , M. Wancura , M. Chwatko , C. Waldron , N. Kalkunte , J. Zoldan , M. Arseneault , A. Elgalad , M. K. Rausch , M. Razavi , E. Cosgriff‐Hernandez , Biomaterials 2025, 317, 123071. 39809077 10.1016/j.biomaterials.2024.123071 PMC12757118

[156] S. Zhuo , A. Tessier , M. Arefi , A. Zhang , C. Williams , S. K. Ameri , npj Flexible Electron. 2024, 8, 49.

[157] S. Lu , J. Luo , L. Qu , K. Li , Y. Li , Q. Zhang , H. Wang , C. Hou , J. Mater. Chem. C 2025, 13, 12287.

[158] J. Wang , B. Wu , P. Wei , S. Sun , P. Wu , Nat. Commun. 2022, 13, 4411. 35906238 10.1038/s41467-022-32140-3 PMC9338060

[159] C. Li , Z. Tan , X. Shi , D. Song , Y. Zhao , Y. Zhang , Z. Zhao , W. Zhang , J. Qi , Y. Wang , X. Wang , Z. Tan , N. Liu , Adv. Sci. 2024, 11, 2406706. 10.1002/advs.202406706 PMC11515898 39206685

[160] Y. Xiong , J. Han , Y. Wang , Z. L. Wang , Q. Sun , Research 2022, 2022. 10.34133/2022/9867378 PMC9414182 36072274

[161] B. Yao , Y. Yan , Q. Cui , S. Duan , C. Wang , Y. Du , Y. Zhao , D. Wu , S. Wu , X. Zhu , T. Hsiai , X. He , Matter 2022, 5, 4407.

[162] F. Zhuo , Z. Ding , Xi Yang , F. Chu , Y. Liu , Z. Gao , H. Jin , S. Dong , X. Wang , J. Luo , Adv. Sci. 2025, 12, 2413141. 10.1002/advs.202413141 PMC11848549 39840613

[163] F. Fallegger , G. Schiavone , S. P. Lacour , Adv. Mater. 2020, 32, 1903904. 10.1002/adma.201903904 31608508

[164] S. Kim , Y. Shin , J. Han , H. J. Kim , S.‐H. Sunwoo , Gels 2024, 10, 614. 39451267 10.3390/gels10100614 PMC11506957

[165] Z. Lu , K. Xu , K. Xiao , Q. Xu , Li Wang , P. Li , J. Zhou , D. Zhao , L. Bai , Y. Cheng , W. Huang , npj Flexible Electron. 2025, 9, 9.

[166] R. Tamate , M. Watanabe , Sci. Technol. Adv. Mater. 2020, 21, 388. 32939164 10.1080/14686996.2020.1777833 PMC7476529

[167] Z. Gao , L. Kong , R. Jin , X. Liu , W. Hu , G. Gao , J. Mater. Chem. C 2020, 8, 11119.

[168] S. Yamada , T. Honda , ACS Mater. Au 2024, 5, 35. 39802151 10.1021/acsmaterialsau.4c00100 PMC11718536

[169] Y. Sun , G. Tian , T. Yang , S. Wang , B. Lan , X. Li , T. Xu , L. Jin , W. Deng , W. Yang , Adv. Funct. Mater. 2025, 35, 2420187.

[170] V. Amoli , J. S. Kim , E. Jee , Y. S. Chung , S. Y. Kim , J. Koo , H. Choi , Y. Kim , D. H. Kim , Nat. Commun. 2019, 10, 4019. 31488820 10.1038/s41467-019-11973-5 PMC6728325

[171] Z. Kong , E. K. Boahen , D. J. Kim , F. Li , J. S. Kim , H. Kweon , S. Y. Kim , H. Choi , J. Zhu , W. Bin Ying , D. H. Kim , Nat. Commun. 2024, 15, 2129. 38459042 10.1038/s41467-024-46334-4 PMC10923942

[172] W. F. Agnew , T. G. H. Yuen , D. B. McCreery , L. A. Bullara , Exp. Neurol. 1986, 92, 162. 3956647 10.1016/0014-4886(86)90132-9

[173] S. Zhao , P. Tseng , J. Grasman , Yu Wang , W. Li , B. Napier , B. Yavuz , Y. Chen , L. Howell , J. Rincon , F. G. Omenetto , D. L. Kaplan , Adv. Mater. 2018, 30, 1800598. 10.1002/adma.201800598 29717798

[174] R. K. Shepherd , P. M. Carter , A. N. Dalrymple , Y. L. Enke , A. K. Wise , T. Nguyen , J. Firth , A. Thompson , J. B. Fallon , J. Neural Eng. 2021, 18, 036021. 10.1088/1741-2552/abe5ba PMC8711780 33578409

[175] Y. Liu , J. Liu , S. Chen , T. Lei , Y. Kim , S. Niu , H. Wang , X. Wang , A. M. Foudeh , J. B.‐H. Tok , Z. Bao , Nat. Biomed. Eng. 2019, 3, 58. 30932073 10.1038/s41551-018-0335-6

[176] J. Bai , D. Liu , X. Tian , Y. Wang , B. Cui , Y. Yang , S. Dai , W. Lin , J. Zhu , J. Wang , A. Xu , Z. Gu , S. Zhang , Sci. Adv. 2024, 10, adl1856. 10.1126/sciadv.adl1856 PMC11029813 38640241

[177] M. K. Islam , A. Rastegarnia , S. Sanei , Signal Processing Techniques for Computational Health Informatics, Springer International Publishing, Cham, Switzerland 2020, 23.

[178] Y. Peng , C. Shi , Y. Zhu , M. Gu , S. Zhuang , PhotoniX 2020, 1, 12.

[179] Y. Baykal , Phys. Scr. 2024, 99, 095513.

[180] M. Zhang , M. Hao , B. Liu , J. Chen , G. Ren , Y. Zhao , J. Guo , L. Zhuang , S. Zhao , Z. Peng , J. Lian , J. Wu , Yi Chen , J. Ma , Q. Lu , Soft Science 2024, 4, 39.

[181] I. You , D. G. Mackanic , N. Matsuhisa , J. Kang , J. Kwon , L. Beker , J. Mun , W. Suh , T. Y. Kim , J. B.‐H. Tok , Z. Bao , U. Jeong , Science 2020, 370, 961. 33214277 10.1126/science.aba5132

[182] T. Yamaguchi , E. Nakahara , S. Koda , J. Phys. Chem. B 2014, 118, 5752. 24802550 10.1021/jp502631q

[183] A. M. Alqudah , Z. Moussavi , Comput. Mater. Contin. 2025, 83, 3753

[184] Z. Feng , Q. He , X. Wang , Y. Lin , J. Qiu , Y. Wu , J. Yang , ACS Appl. Mater. Interfaces 2023, 15, 6217. 36691890 10.1021/acsami.2c21885

[185] M. Kim , S. Hong , R. Khan , J. J. Park , J. B. In , S. H. Ko , Small 2025, 21, 2405301. 10.1002/smll.202405301 39610205

[186] S. G. Lee , K. J. Yu , S. M. Won , J.‐Y. Yoo , Int. J. Extr. Manuf. 2025, 7, 042003.

[187] Z. Zhang , Z. Chen , T. Liu , L. Zhang , Chem. Sci. 2025, 16, 7963. 40201170 10.1039/d5sc01609f PMC11973721

[188] Y. R. Jeong , G. Lee , H. Park , J. S. Ha , Acc. Chem. Res. 2018, 52, 91. 30586283 10.1021/acs.accounts.8b00508

[189] C. Sun , J. Luo , T. Jia , C. Hou , Y. Li , Q. Zhang , H. Wang , Chem. Eng. J. 2022, 431, 134012,

[190] Q. Xiao , Y. Gong , H. Zhou , Y. Zhang , Q. Shen , X. Sun , Carbohydr. Polym. 2025, 367, 124038. 40817559 10.1016/j.carbpol.2025.124038

[191] M. T. Khan , T. U. Rehman , L. A. Shah , H. Yoo , Int. J. Adhes. Adhes. 2025, 367, 104088.

[192] Y. Guo , R. Liang , J. Zhang , H. Yuan , J. Wang , Y. Le , New J. Chem. 2025, 49, 15119.

[193] Y. Li , N. Li , N. De Oliveira , S. Wang , Matter 2021, 4, 1125.

[194] J. Qu , K. Xie , S. Chen , X. He , Y. Wang , M. Chamberlin , Xi Zhao , G. Zhu , C. Xu , P. Shi , Sci. Adv. 2024, 10, adq9207. 10.1126/sciadv.adq9207 PMC11584000 39576849

[195] T. Chu , Y. Xiao , H. Lai , L. Shi , Y. Cheng , J. Sun , Z. Pang , S. Cheng , K. Zhao , Z. Gao , R. Wang , ACS Nano 2025, 19, 18729. 40336176 10.1021/acsnano.5c03336

[196] L. Qian , F. Jin , T. Li , Z. Wei , X. Ma , W. Zheng , N. Javanmardi , Z. Wang , J. Ma , C. Lai , W. Dong , T. Wang , Z‐Qi Feng , Adv. Mater. 2024, 36, 2406636. 10.1002/adma.202406636 39148152

[197] I. Lerman , Y. Bu , R. Singh , H. A. Silverman , A. Bhardwaj , A. J. Mann , A. Widge , J. Palin , C. Puleo , H. Lim , Bioelectronic Medicine 2025, 11, 1. 39833963 10.1186/s42234-024-00163-4 PMC11748337

[198] Y. Lee , Y. Liu , D.‐G. Seo , J. Y. Oh , Y. Kim , J. Li , J. Kang , J. Kim , J. Mun , A. M. Foudeh , Z. Bao , T.‐W. Lee , Nat. Biomed. Eng. 2023, 7, 511. 35970931 10.1038/s41551-022-00918-x

[199] Lu Zhang , D. Jiang , T. Dong , R. Das , D. Pan , C. Sun , Z. Wu , Q. Zhang , C. Liu , Z. Guo , Chem. Rec. 2020, 20, 948. 32657539 10.1002/tcr.202000041

[200] W. Zhan , H. Zhang , X. Lyu , Z.‐Z. Luo , Y. Yu , Z. Zou , Sci. China Mater. 2023, 66, 1539.

[201] L. Lu , Xu Liu , P. Gu , Z. Hu , X. Liang , Z. Deng , Z. Sun , X. Zhang , X. Yang , J. Yang , G. Zu , J. Huang , Nat. Commun. 2025, 16, 3831. 40268969 10.1038/s41467-025-59240-0 PMC12019246

[202] Y.‐S. Lim , J. H. Kim , J. Kim , M. Hoang , W. Kang , M. Koh , W. H. Choi , S. Park , U. Jeong , D. H. Kim , S.‐M. Park , Nat. Commun. 2025, 16, 4115. 40316532 10.1038/s41467-025-59436-4 PMC12048617

[203] M. Khalili , H. GholamHosseini , A. Lowe , M. M. Y. Kuo , Med. Biol. Eng. Comput. 2024, 62, 3599. 39031328 10.1007/s11517-024-03165-1 PMC11568998

[204] J. Lee , J. Heo , W. Lee , Y. Lim , Y. Kim , K. Park , Sensors 2014, 14, 14732. 25120162 10.3390/s140814732 PMC4179047

[205] B. Sanchez‐Lengeling , A. Aspuru‐Guzik , Science 2018, 361, 360. 30049875 10.1126/science.aat2663

[206] W. Chen , A. Iyer , R. Bostanabad , Engineering 2022, 10, 89.

[207] J. Schaeffer , P. Gasper , E. Garcia‐Tamayo , R. Gasper , M. Adachi , J. Pablo Gaviria‐Cardona , S. Montoya‐Bedoya , A. Bhutani , A. Schiek , R. Goodall , R. Findeisen , R. D. Braatz , S. Engelke , J. Electrochem. Soc. 2023, 170, 060512.

[208] A. M. Oliveira , L. Coelho , E. Carvalho , M. J. Ferreira‐Pinto , R. Vaz , P. Aguiar , J. Neurol. 2023, 270, 5313. 37530789 10.1007/s00415-023-11873-1 PMC10576725

[209] H. Qiu , Z.‐Y. Sun , npj Comput. Mater. 2024, 10, 273.

[210] H. Tran , R. Gurnani , C. Kim , G. Pilania , Ha‐K Kwon , R. P. Lively , R. Ramprasad , Nat. Rev. Mater. 2024, 9, 866.

[211] B. Albakri , A. T. S. Diniz , P. Benner , T. Muth , S. Nakajima , M. Favaro , A. Kister , Electrochim. Acta 2024, 496, 144474.

[212] A. S. Chandrabhatla , I. J Pomeraniec , T. M. Horgan , E. K. Wat , A. Ksendzovsky , NPJ Digital Medicine 2023, 6, 79. 37106034 10.1038/s41746-023-00779-x PMC10140375

